# Rapid Recapitulation of Nonalcoholic Steatohepatitis upon Loss of Host Cell Factor 1 Function in Mouse Hepatocytes

**DOI:** 10.1128/MCB.00405-18

**Published:** 2019-02-15

**Authors:** Shilpi Minocha, Dominic Villeneuve, Viviane Praz, Catherine Moret, Maykel Lopes, Danièle Pinatel, Leonor Rib, Nicolas Guex, Winship Herr

**Affiliations:** a Center for Integrative Genomics, Génopode, University of Lausanne, Lausanne, Switzerland; b Swiss Institute of Bioinformatics, Génopode, University of Lausanne, Lausanne, Switzerland

**Keywords:** HCF-1, Hcfc1, NAFLD, NASH, PGC1alpha, chromatin, gene expression, steatosis

## Abstract

Host cell factor 1 (HCF-1), encoded by the ubiquitously expressed X-linked gene *Hcfc1*, is an epigenetic coregulator important for mouse development and cell proliferation, including during liver regeneration. We used a hepatocyte-specific inducible *Hcfc1* knockout allele (called *Hcfc1*^hepKO^) to induce HCF-1 loss in hepatocytes of hemizygous *Hcfc1*^hepKO/Y^ males by 4 days. In heterozygous *Hcfc1*^hepKO/+^ females, owing to random X-chromosome inactivation, upon *Hcfc1*^hepKO^ allele induction, a 50/50 mix of HCF-1-positive and -negative hepatocyte clusters is engineered. The livers with *Hcfc1*^hepKO/Y^ hepatocytes displayed a 21- to 24-day terminal nonalcoholic fatty liver (NAFL), followed by nonalcoholic steatohepatitis (NASH) disease progression typical of severe NAFL disease (NAFLD). In contrast, in livers with heterozygous *Hcfc1*^hepKO/+^ hepatocytes, HCF-1-positive hepatocytes replaced HCF-1-negative hepatocytes and revealed only mild NAFL development. Loss of HCF-1 led to loss of PGC1α protein, probably owing to its destabilization, and deregulation of gene expression, particularly of genes involved in mitochondrial structure and function, likely explaining the severe *Hcfc1*^hepKO/Y^ liver pathology. Thus, HCF-1 is essential for hepatocyte function, likely playing both transcriptional and nontranscriptional roles. These genetically engineered loss-of-HCF-1 mice can be used to study NASH as well as NAFLD resolution.

## Abstract (precis)

Host cell factor 1 (HCF-1), encoded by the ubiquitously expressed X-linked gene *Hcfc1*, is an epigenetic coregulator important for mouse development and cell proliferation, including during liver regeneration. We used a hepatocyte-specific inducible *Hcfc1* knockout allele (called *Hcfc1*^hepKO^) to induce HCF-1 loss in hepatocytes of hemizygous *Hcfc1*^hepKO/Y^ males by 4 days.

## INTRODUCTION

Nonalcoholic fatty liver disease (NAFLD) encompasses a wide range of liver conditions caused by excessive fat accumulation or steatosis within hepatocytes (1–5). NAFLD consists of two phases: a less severe state of nonalcoholic fatty liver (NAFL), where patients develop hepatic steatosis, followed in a minority of patients by a more severe nonalcoholic steatohepatitis (NASH) condition associated with steatosis, followed by fibrosis to cirrhosis and even hepatocellular carcinoma (HCC) (6–8). Although steatosis is considered a hallmark of NAFLD, it is not clearly known how NAFLD transitions from the simple steatosis of NAFL to NASH, although mitochondrial malfunction has been implicated in a two-hit steatosis-followed-by-oxidative-stress hypothesis (9, 10).

Surprisingly, despite the current large increases in the number of NAFLD patients in most developed countries (11), very little is known about the mechanisms of this disease, in part owing to the lack of suitable animal models for its study. Current models include genetic (e.g., PTEN liver knockout), nutritional (e.g., high fat and methionine and choline deficient [MCD]), and combinations of the two (e.g., *db/db* mice on an MCD diet), but none of these recapitulate the entire NAFLD process (12–14) or take about 24 weeks to develop (15). Hence, alternative animal models would aid in elucidating the mechanism of disease progression and identifying pharmacotherapeutic options for both its prevention and cure. Here, we show that hepatocyte-specific disruption of HCF-1 function leads to a rapid recapitulation of NAFLD, including NASH.

HCF-1, encoded by the X-linked *HCFC1* and *Hcfc1* genes in human and mice, respectively, is a conserved metazoan transcriptional coregulator that binds to the transcriptional start sites of many genes (16, 17) and physically links sequence-specific DNA-binding transcription factors with chromatin-modifying enzymes, such as mixed-lineage leukemia (MLL) and Set1 histone H3 lysine 4 (H3K4) methyltransferases (for a review, see reference 18).

HCF-1 in humans and mice has been implicated in both proliferative and nonproliferative cell functions. It is important for cell proliferation in cell culture and in mice during both embryogenesis and liver regeneration (19–23). Human clinical studies of X-linked diseases, including mental disability and cobalamin disorder, have linked the *HCFC1* gene to nonproliferative functions (24–27). Furthermore, stabilization of PGC-1α by HCF-1 has been shown to promote glucose production in hepatocytes, and knockdown of HCF-1 improves glucose homeostasis in diabetic mice (28).

Here, we investigated the long-term role of HCF-1 in the resting mouse liver by induced hepatocyte-specific inactivation of its cognate gene, *Hcfc1*.

## RESULTS

### Rapid induction of loss of *Hcfc1* expression in adult liver hepatocytes.

To probe HCF-1 function in adult livers, we induced, via tamoxifen-dependent Cre-mediated loxP site recombination, deletion of *Hcfc1* exons 2 and 3 in hepatocytes of hemizygous males carrying the *Albumin-Cre-ERT2^tg^* transgene (generating the *Hcfc1*^hepKO^ allele) as described previously (22). Identically treated *Hcfc1*^lox/Y^ males lacking the hepatocyte-specific *Albumin-Cre-ERT2* transgene served as controls, particularly for the prominent effects of tamoxifen treatment alone. Tamoxifen treatment of heterozygous *Hcfc1*^lox/+^ females carrying the *Albumin-Cre-ERT2* transgene leads to patches of HCF-1-positive and -negative cells owing to whether the *Hcfc1*^hepKO^ allele is generated on the inactive or active X chromosome, respectively (22).

**FIG 1:**
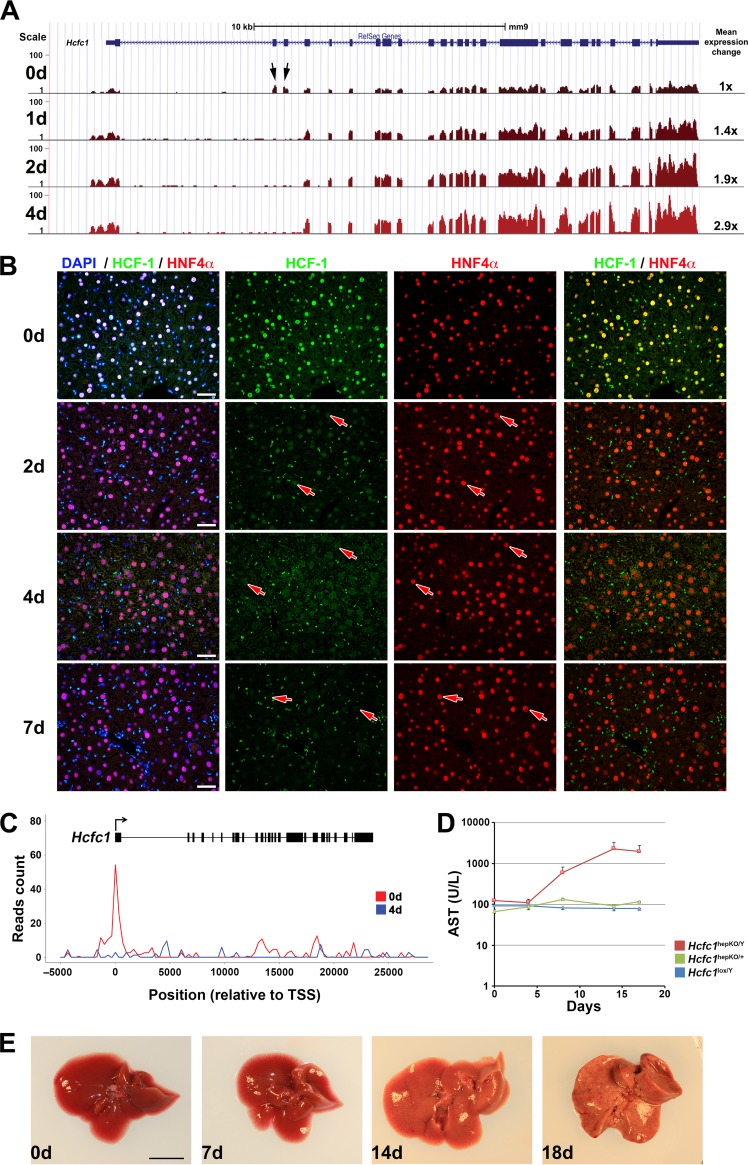
*Hcfc1*^hepKO/Y^ males display rapid loss of wild-type *Hcfc1* expression in hepatocytes and subsequent liver pathology. (A) View of the reads mapped on the 26 exons of the *Hcfc1* gene, shown in blue at the top, in control (day 0 [0d]) liver and *Alb-Cre-ERT2*^tg^; *Hcfc1*^hepKO/Y^ livers 1, 2, and 4 days (1d, 2d, and 4d, respectively) after tamoxifen treatment according to RNA-Seq analysis. Arrows point to the reads observed at *Hcfc1* exons 2 and 3. The total relative *Hcfc1* transcript expression level is listed to the right. (B) Immunofluorescence analysis of paraffin-embedded sections from control (0d) liver and *Alb-Cre-ERT2*^tg^; *Hcfc1*^hepKO/Y^ livers 2, 4, and 7 days after tamoxifen treatment and stained with 4’,6-diamidino-2-phenylindole (DAPI) (blue) together with antibodies against HCF-1 (green) and HNF4α (red). Arrows point to hepatocytes. Scale bars, 50 μm. (C) Image of HCF-1 ChIP-Seq reads mapped onto the *Hcfc1* gene from control (0d) and *Alb-Cre-ERT2*^tg^; *Hcfc1*^hepKO/Y^ livers 4 days after tamoxifen treatment (4d). TSS, transcription start site. (D) AST levels in control *Hcfc1*^lox/Y^ (blue; *n* = 3/time point), *Alb-Cre-ERT2*^tg^; *Hcfc1*^hepKO/+^ (green; *n* = 3/time point), and *Alb-Cre-ERT2*^tg^; *Hcfc1*^hepKO/Y^ (red; *n* = 3/time point) livers at 0, 4, 8, 14, and 17 days after tamoxifen treatment. (E) Macroscopic comparison of control liver (0d) and tamoxifen-treated knockout *Alb-Cre-ERT2*^tg^; *Hcfc1*^hepKO/Y^ livers after 7, 14, and 18 days. Scale bar, 1 cm.

To determine the time course of tamoxifen activation of the *Hcfc1*^hepKO^ allele, we assayed *Hcfc1* transcripts by RNA-Seq of poly(A)-selected RNA (Fig. 1A; see also Fig. S1 in the supplemental material) and levels of HCF-1 protein by immunofluorescence (Fig. 1B). Whereas RNA-Seq tags were readily detected for all *Hcfc1* exons prior to tamoxifen treatment (0d samples in Fig. 1A and Fig. S1A to C), in *Hcfc1*^hepKO/Y^ males, exon 2 and 3 tags rapidly disappeared, as early as day 1 after treatment (Fig. 1A, arrows, and Fig. S1C and Table S1), and tags containing a novel exon 1 to exon 4 splice junction appeared (Table S1). In contrast, there was no change in *Hcfc1* expression in tamoxifen-treated control *Hcfc1*^lox/Y^ livers (Fig. S1A and Table S1). In *Hcfc1*^hepKO/+^ heterozygous females, the loss of exon 2 and 3 tags was less drastic, yet the exon 1 to 4 splice junction tags diagnostic of *Hcfc1*^hepKO^ allele generation were evident (Fig. S1B and Table S1), indicating heterozygous *Hcfc1*^hepKO^ allele generation.

The rapid disappearance of the wild-type *Hcfc1* transcript after tamoxifen treatment indicates that the synthesis of HCF-1 in hepatocytes is likewise rapidly blocked. In cultured cells, HCF-1 has a long half-life (29). Consistent with such stability, progressive and significant hepatocyte-specific (i.e., HNF4α positive) reduction in HCF-1 protein levels was seen in knockout males from day 2 onwards with an apparent complete absence by day 7 (Fig. 1B, arrows). In contrast, non-hepatocyte HNF4α-negative cells continued to show high HCF-1 levels (Fig. 1B). Loss of HCF-1 was also detected at its many genomic binding sites (see below). For example, here (Fig. 1C) we show HCF-1 bound to the *Hcfc1* promoter at day 0, which is already lost by day 4. Interestingly, as hepatocyte HCF-1 levels decreased in *Hcfc1*^hepKO/Y^ males and *Hcfc1* promoter-bound HCF-1 disappeared, the levels of deleted *Hcfc1*^hepKO^ transcripts increased (Fig. 1A and Fig. S1), suggesting that the *Hcfc1* gene is under negative HCF-1 autoregulation.

### Loss of hepatocyte *Hcfc1* expression leads to liver pathology.

As a first measure of liver-specific pathology upon *Hcfc1*^hepKO^ allele generation, we measured levels of the liver injury marker aspartate aminotransferase (AST) in serum (Fig. 1D). Whereas AST levels remained stable in control *Hcfc1*^lox/Y^ males and initially in *Hcfc1*^hepKO/Y^ males, by 8 days there was a large increase in the *Hcfc1*^hepKO/Y^ males (Fig. 1D) following loss of HCF-1 protein (Fig. 1B and C). In contrast, in *Hcfc1*^hepKO/+^ heterozygous females, there was only a brief marginal increase in AST levels at 8 days after tamoxifen treatment (Fig. 1D), indicating only a mild pathological response to heterogeneous HCF-1 loss in hepatocytes.

With time, the *Hcfc1*^hepKO/Y^ male livers became larger and paler as well as visibly fatty (Fig. 1E), and the liver-to-body weight ratio increased considerably by 18 days after tamoxifen treatment (Fig. S2B). Nevertheless, the body weight of *Hcfc1*^hepKO/Y^ males remained largely unaffected through 14 days after tamoxifen treatment, with a marginal decrease observed from 15 days onward (Fig. S3A). Such overt pathologies were not observed in heterozygous *Hcfc1*^hepKO/+^ female mice or livers (Fig. S2A and C). Furthermore, while *Hcfc1*^hepKO/+^ females appeared healthy and survived, the *Hcfc1*^hepKO/Y^ males appeared ill by 18 days after tamoxifen treatment and did not survive beyond 21 to 24 days after tamoxifen treatment.

To probe further any effects of universal hepatocyte loss of HCF-1 in *Hcfc1*^hepKO/Y^ males or of heterogeneous hepatocyte HCF-1 loss in *Hcfc1*^hepKO/+^ females, we performed carbohydrate metabolism tests (Fig. S3B to G) and measured liver metabolite concentrations (Fig. S4). Compared to control males, in a glucose tolerance test, the *Hcfc1*^hepKO/Y^ males did not show any initial difference in blood glucose levels and, moreover, retained a healthy response (Fig. S3B), as did control and heterozygous *Hcfc1*^hepKO/+^ females (Fig. S3C). Consistent with these results, there were no significant differences in basal or glucose-stimulated insulin secretion levels between control and *Hcfc1*^hepKO/Y^ males (Fig. S3F), and they likewise responded similarly to an insulin tolerance test (Fig. S3G), indicating that the *Hcfc1*^hepKO/Y^ males are insulin sensitive.

In contrast, in pyruvate tolerance tests, the *Hcfc1*^hepKO/Y^ males were defective for gluconeogenesis (Fig. S3D), a defect that was significantly less pronounced in heterozygous *Hcfc1*^hepKO/+^ females (Fig. S3E). In accordance with this *Hcfc1*^hepKO/Y^ gluconeogenesis defect, we observed a reduction in the levels of dihydroxyacetone phosphate (DHAP) and 3-phosphoglycerate (3-PGA), two intermediates in the gluconeogenesis pathway, in *Hcfc1*^hepKO/Y^ versus control males (Fig. S4A) and an overall increase in glycogenolysis intermediates (i.e., glucose-1-phosphate, glucose-6-phosphate, and fructose-6-phosphate; referred to here as Hex-P) (Fig. S4A).

Hence, biochemical, morphological, and metabolic analyses all indicated that *Hcfc1*^hepKO/Y^ males succumb to liver damage culminating in death, whereas *Hcfc1*^hepKO/+^ females with heterogeneous HCF-1 loss display transient alterations that return to normalcy.

### *Hcfc1*^hepKO/Y^ males display characteristic NAFL progression to NASH of NAFLD.

**FIG 2:**
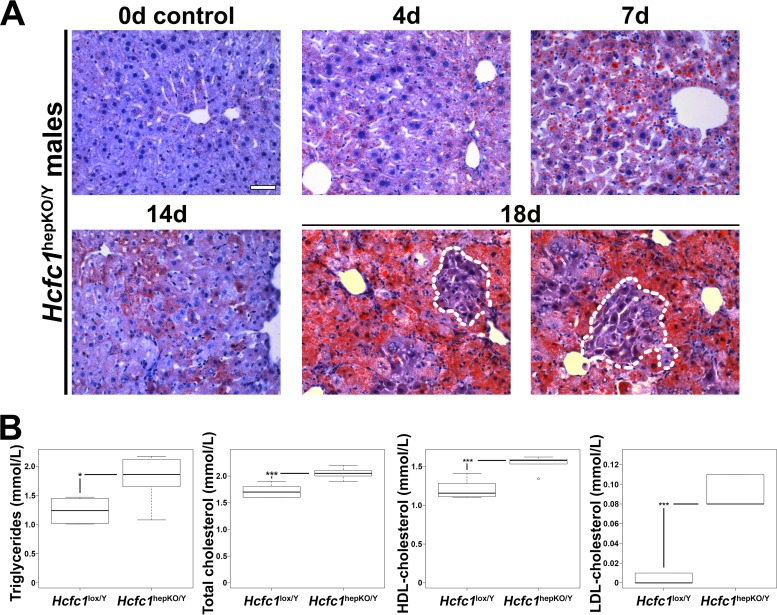
*Hcfc1*^hepKO/Y^ males display increased steatosis. (A) Steatosis was measured by Oil Red O staining of cryosections from control liver (0d) and tamoxifen-treated *Alb-Cre-ERT2*^tg^; *Hcfc1*^hepKO/Y^ livers 4, 7, 14, and 18 days after treatment. Dotted lines outline hepatocyte clusters with reduced levels of fat accumulation. Scale bar, 50 μm. (B) Box plots of circulating triglyceride, total cholesterol, and HDL and LDL cholesterol levels in control *Hcfc1*^lox/Y^ (*n* = 6) compared to *Alb-Cre-ERT2*^tg^; *Hcfc1*^hepKO/Y^ (*n* = 6) livers 4 days after tamoxifen treatment. The differences between the levels of triglycerides (*P* value of 0.02), total cholesterol (*P* value of 4.2 × 10^−4^), and HDL (*P* value of 4.6 × 10^−4^) and LDL (*P* value of 1.13 × 10^−5^) cholesterol in control and knockout livers were significant.

To define the nature of the liver damage, we performed comparative histology of the control and *Hcfc1*^hepKO/Y^ males. As the *Hcfc1*^hepKO/Y^ male livers appeared pale and fatty (Fig. 1E), we first performed Oil Red O staining to probe for steatosis. Indeed, the *Hcfc1*^hepKO/Y^ males displayed increased steatosis by 4 days (Fig. 2A). Lipid droplets were widespread and increased over time. Consistent with increased lipid content, metabolomic analyses at 7 days revealed elevated levels of fatty acid oxidation intermediates in the form of short-, medium-, and long-chain acylcarnitines (Fig. S4B). By 18 days, the steatosis was particularly severe, yet we noted clusters of hepatocytes with reduced fat content near hepatic veins, as discussed further below (Fig. 2A, outlined areas). The increase in liver fat content was reflected systemically by increased levels of circulating blood serum triglycerides and high-density lipoprotein (HDL), low-density lipoprotein (LDL), and total cholesterol already by 4 days (Fig. 2B). These increased lipid-associated properties are typical of NAFLD progression (2).

Also associated with hepatocellular injury, hyperlipidemia, and NAFLD progression is faulty bile acid secretion and amino acid accumulation (30, 31). Indeed, *Hcfc1*^hepKO/Y^ males possessed increased concentrations of a number of bile acids at 7 days after tamoxifen treatment (Fig. S4C). Furthermore, several amino acids (e.g., glutamine, glutamate, histidine, and serine) were elevated in *Hcfc1*^hepKO/Y^ males at 7 days (Fig. S4D).

**FIG 3:**
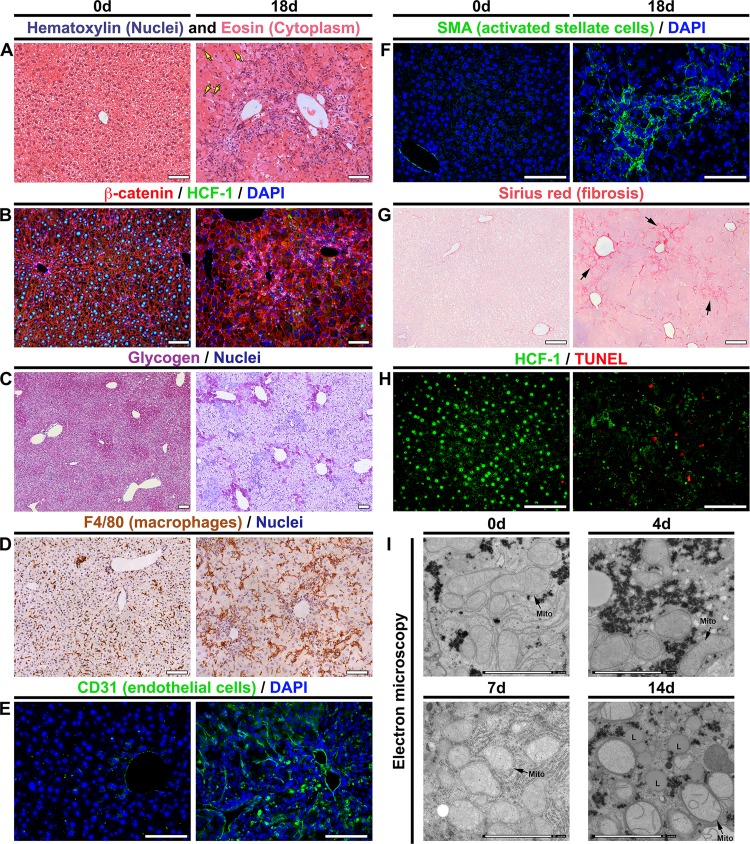
*Hcfc1*^hepKO/Y^ males display characteristics typical of NASH by 18 days after tamoxifen treatment. (A to H) Comparison of paraffin-embedded sections (A to D and F to H) and cryosections (E) of control livers (0d; left) and *Alb-Cre-ERT2*^tg^; *Hcfc1*^hepKO/Y^ livers 18 days after tamoxifen treatment (right). (A) Hematoxylin (blue) and eosin (pink) staining. Arrows point to hepatocytes with various nuclear sizes. (B) Sections stained with DAPI (blue) together with β-catenin (red) and HCF-1 (green) antibodies. (C) Hepatic glycogen visualized by PAS staining (purple). The sections were also stained with hematoxylin (blue). (D) DAB immunostaining for macrophage marker F4/80 (brown). The sections were also stained with hematoxylin (blue). (E) Cryosections stained with DAPI (blue) and CD-31 antibody (green). (F) Sections stained with DAPI (blue) and smooth-muscle α-actin (SMA; green) antibody. (G) Sections stained with Sirius red. The arrows point to collagen fibers. (H) TUNEL assay of apoptotic cells (red) costained with HCF-1 antibody (green). TUNEL-positive apoptotic cells are shown in red. (I) Electron microscopic images of control liver (0d) and knockout *Alb-Cre-ERT2*^tg^; *Hcfc1*^hepKO/Y^ male liver samples 4, 7, and 14 days after tamoxifen treatment. Scale bars: 100 μm (A to H) and 2 μm (I).

The aforementioned metabolic disorders indicate a severe and rapid destabilization of hepatocyte function upon the loss of HCF-1. To characterize the course of the pathology, we performed histology for an array of markers affected by NAFLD progression. Figure 3 compares unaffected livers at day 0 and highly diseased livers at 18 days after *Hcfc1*^hepKO/Y^ induction; examples of intermediate states for many of the histological analyses are shown in Fig. 5 to 8. Hematoxylin and eosin (H&E) staining (Fig. 3A and Fig. S5) showed (i) marked tissue disorganization and breakdown of extracellular matrix by 7 days, (ii) increased inflammatory cell infiltration, (iii) heterogeneous hepatocyte nuclear sizes, referred to as nuclear anisocytosis or dysplasia (arrows), and (iv) often a ballooning phenotype of hepatocytes; all of these signs are typical of extensive liver injury. Coimmunostaining for the plasma membrane marker β-catenin and HCF-1 (Fig. 3B) revealed, in addition to many of the aforementioned phenotypes, the general restriction of HCF-1 to nonhepatocytes at 18 days.

Hepatic glycogen content is markedly reduced in carbon tetrachloride-induced rat cirrhosis (32) and human cirrhotic patients (32). Periodic acid-Schiff (PAS) staining for glycogen in the *Hcfc1*^hepKO/Y^ male livers (Fig. 3C and Fig. S6) showed a progressive decline in glycogen content beginning by 7 days with a drastic reduction observed at 18 days, consistent with hepatocyte dysfunction.

Inflammation, transformation of the liver sinusoidal endothelium into a continuous vascular type, and activation of stellate cells resulting in fibrosis are key hallmarks of NASH during late-stage NAFLD (reviewed in reference 2). Consistent with the *Hcfc1*^hepKO/Y^ male disease progression reflecting NAFLD, we observed (i) a progressive increase in the macrophage inflammatory marker F4/80 by 7 days (Fig. S7) and major clusters of F4/80-positive macrophages throughout at 18 days (Fig. 3D); (ii) extensive vascular endothelium development (i.e., CD31 or PECAM endothelial cell marker positive) (Fig. 3E); and (iii) both activated stellate cells (i.e., smooth muscle α-actin [SMA]-positive cells) (Fig. 3F) at 18 days and progressive increase in fibrosis (i.e., Sirius red positive) beginning around 9 days (Fig. 3G and Fig. S8). In addition to these aforementioned NASH hallmarks, we observed significant cell death (i.e., terminal deoxynucleotidyltransferase-mediated dUTP-biotin nick end labeling [TUNEL] positive) at 18 days (Fig. 3H).

As mitochondrial malfunction is associated with NAFLD, we examined mitochondrial structure in liver biopsy specimens from *Hcfc1*^hepKO/Y^ males by electron microscopy, as shown in Fig. 3I and Fig. S9. Consistent with the aforementioned results with PAS (Fig. 3C), Oil Red O (Fig. 2A), and Sirius red (Fig. 3G) staining and under the lower electron microscopy magnification images shown in Fig. S9, glycogen stores decreased and lipid deposits and collagen fiber increased progressively over the 14-day time course. In parallel, although there was no evident decrease in the number of mitochondria, they began to display reduced numbers of cristae after 4 days, which became more evident at 7 to 14 days after tamoxifen treatment in the *Hcfc1*^hepKO/Y^ males (Fig. 3I). Indeed, some mitochondria completely lacked cristae and were swollen and spherical in shape after 14 days of tamoxifen treatment (Fig. 3I and Fig. S9). At the same time, there were multiple membranes around the mitochondria (Fig. S9, arrowheads). The results of these ultrastructural analyses are consistent with those observed in liver biopsy samples from NAFLD/NASH patients (33, 34).

In summary, the *Hcfc1*^hepKO/Y^ males recapitulate several physiological, metabolic, histological, and ultrastructural features of NAFL progression to NASH in NAFLD: increased steatosis, tissue disorganization, abnormal hepatocellular structure, metabolic disorders, inflammation, sinusoidal vascularization, fibrosis, mitochondrial defects, and increased cell death.

### PGC1α protein, but not corresponding transcript levels, decreases upon *Hcfc1* inactivation.

As described in the introduction, HCF-1 is implicated in both stabilization of PGC1α, thereby promoting gluconeogenesis (28), and direct transcriptional control through binding to a multitude of transcriptional regulators at transcriptional start sites (18). Disruption of both PGC1α stabilization and transcriptional control could be implicated in the NAFLD progression observed upon loss of HCF-1 in hepatocytes. Thus, we probed disruption of each of these activities.

**FIG 4:**
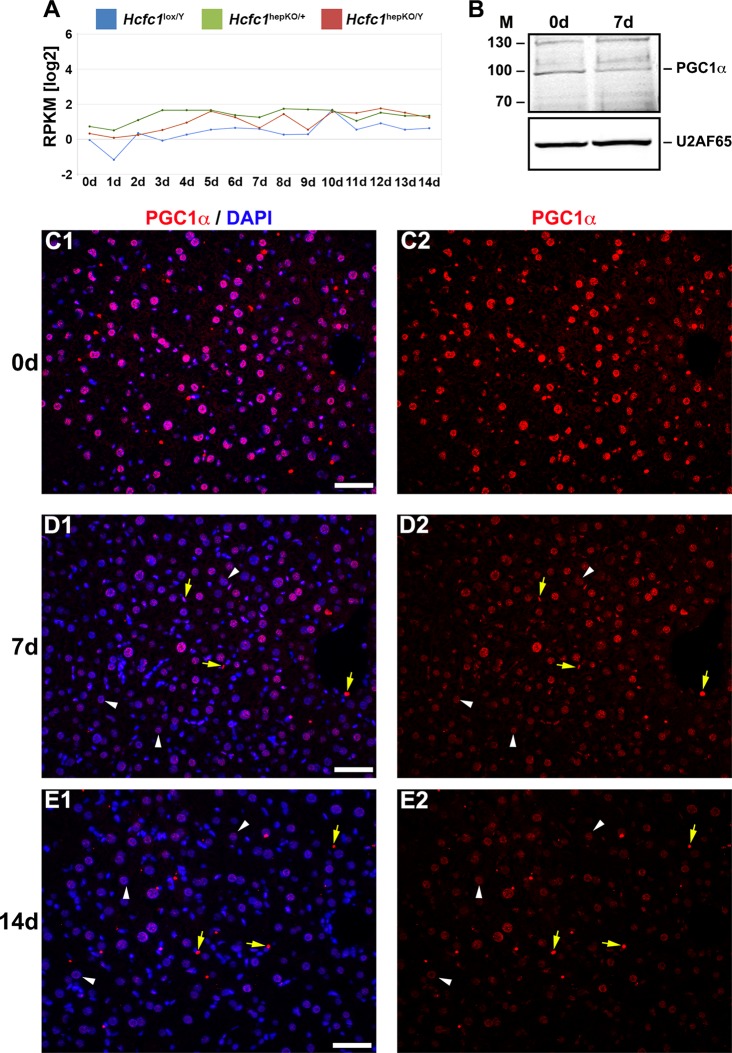
*Hcfc1*^hepKO/Y^ male livers display reduced PGC1α protein levels. (A) The total *Ppargc1a* transcript expression level in control *Hcfc1*^lox/Y^ (blue), *Alb-Cre-ERT2*^tg^; *Hcfc1*^hepKO/+^ (green), and *Alb-Cre-ERT2*^tg^; *Hcfc1*^hepKO/Y^ (red) livers. RPKM, reads per kilobase of transcript per million mapped reads. (B) Immunoblotting with anti-PGC1α antibody and anti-U2AF65 antibody as a loading control with liver lysates of samples collected from control (0d) liver and *Alb-Cre-ERT2*^tg^; *Hcfc1*^hepKO/Y^ male livers 7 days after tamoxifen treatment. M, molecular size (in kilodaltons). (C to E) Immunofluorescence analysis of paraffin-embedded sections from control (0d) liver (C) and *Alb-Cre-ERT2*^tg^; *Hcfc1*^hepKO/Y^ male livers 7 (D) and 14 (E) days after tamoxifen treatment stained with DAPI (blue) and PGC1α (red) antibody (left) or PGC1α antibody alone (right). Arrowheads point to hepatocyte nuclei, and arrows point to non-hepatocyte cell nuclei. Scale bars, 50 μm.

RNA-Seq analysis showed that PGC1α-encoding mRNA levels in the liver were low but not perturbed by the loss of HCF-1 (Fig. 4A). In contrast, PGC1α protein levels decreased, as evidenced by the partial overall loss at 7 days in an immunoblot (Fig. 4B) and the sharp progressive reduction observed only in hepatocytes in immunostained samples at 7 and 14 days (compare Fig. 4C to E), a result consistent with a cell-autonomous effect of loss of HCF-1 in hepatocytes on PGC1α-protein levels. Thus, consistent with Ruan et al. (28), loss of HCF-1 leads to loss of PGC1α protein. Owing to the multiple roles of PGC1α in hepatocyte metabolism, including mitochondrial function and gluconeogenesis, this destabilization of PGC1α may play a role in the NAFLD progression observed in *Hcfc1*^hepKO/Y^ males. Nevertheless, loss of PGC1α does not lead to the severe NASH or lethality observed upon loss of HCF-1 (35–37). Thus, HCF-1 likely plays additional PGC1α-independent roles that are disrupted in the *Hcfc1*^hepKO/Y^ males. To probe for these activities, we turned to an analysis of the effects of loss of HCF-1 on gene expression.

### Gene expression changes upon hepatocyte-specific loss of HCF-1.

**FIG 5:**
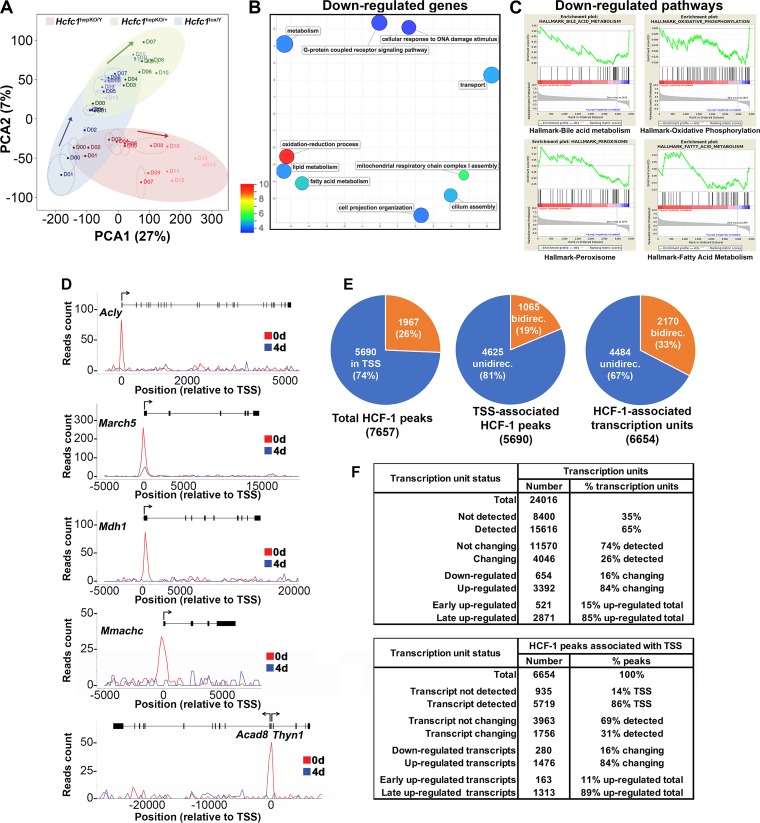
RNA-Seq and ChIP-Seq analyses of control and *Hcfc1*^hepKO/Y^ male livers identify extensive alterations in gene expression upon loss of HCF-1. (A) Two-dimensional PCA plot of 15,616 expressed genes for control *Hcfc1*^lox/Y^ male (day 0 [D00] to D14; blue), *Alb-Cre-ERT2*^tg^; *Hcfc1*^hepKO/+^ (D00 to D13, except D02 and D09; green), and *Alb-Cre-ERT2*^tg^; *Hcfc1*^hepKO/Y^ (D00 to D14; red) livers after tamoxifen treatment. The coordinates of replicate samples were averaged and displayed as single dots, with two-dimensional standard deviations indicated with broken lines. Paths followed by the control male (blue), heterozygous female (green), and knockout male (red) liver transcriptomes are highlighted by ovals and arrows. (B) REVIGO display of functional enrichment analysis of 654 downregulated transcripts in *Hcfc1*^hepKO/Y^ livers. The *P* value of the color key is given. (C) GSEA results for the 654 downregulated transcripts in *Hcfc1*^hepKO/Y^ livers ranked by fold change (Hcfc1^hepKO/Y^ versus Hcfc1^lox/Y^) for the four most statistically significant HALLMARK pathways. (D) Images of the HCF-1 ChIP-Seq profiles mapped on the *Acly*, *March5*, *Mdh1*, *Mmachc*, and neighboring bidirectional *Acad8* and *Thyn1* genes in control (0d; red) liver and *Alb-Cre-ERT2*^tg^; *Hcfc1*^hepKO/Y^ (blue) male livers 4 days after tamoxifen treatment. (E) Pie charts displaying (from left to right) (i) the ratio of TSS- versus non-TSS-associated (±250 bp) HCF-1 peaks; (ii) the ratio of TSS-associated HCF-1 peaks with either a single or double (generally bidirectional) TSS; and (iii) the ratio of HCF-1-associated unidirectional or bidirectional transcription units. (F) Summary of the RNA-Seq and ChIP-Seq analyses.

To examine the effects of HCF-1 loss on gene expression, we compared the RNA-Seq results of *Hcfc1*^hepKO/Y^ male livers to those for control *Hcfc1*^lox/Y^ male and *Hcfc1*^hepKO/+^ heterozygous female livers. Figure 5A shows the results of principal component analysis (PCA) of aggregated RNA-Seq results over the course of 14 days after tamoxifen treatment. Before tamoxifen treatment at day 0 (D00), the *Alb-Cre-ERT2*^tg^; *Hcfc1*^lox/Y^ and *Hcfc1*^lox/Y^ males are similarly positioned on the PCA plot, whereas the *Alb-Cre-ERT2*^tg^; *Hcfc1*^lox/+^ heterozygous females are clearly separated, probably owing to sexual dimorphism. Nevertheless, after tamoxifen treatment, the *Hcfc1*^lox/Y^ males and *Hcfc1*^hepKO/+^ females follow a similar upward PCA trajectory in this plot. The similarity in gene expression pattern between the *Hcfc1*^lox/Y^ males and *Hcfc1*^hepKO/+^ females over the time course of 14 days is consistent with the lack of pronounced *Hcfc1*^hepKO/+^ female pathology (Fig. 1D). In contrast, in the plot, the *Hcfc1*^hepKO/Y^ males follow a distinctly different PCA trajectory away (right and downward) from the control *Hcfc1*^lox/Y^ males and *Hcfc1*^hepKO/+^ females. Thus, loss of HCF-1 causes significant changes in liver gene expression.

To characterize these changes, we identified the gene transcripts that are either down- or upregulated in *Hcfc1*^hepKO/Y^ males compared to control *Hcfc1*^lox/Y^ males. In this way, we eliminated many genes affected solely by the tamoxifen treatment. Thus, we identified 654 downregulated transcripts (Table S2) and 3,392 upregulated transcripts. Of the 3,392 upregulated transcripts, clustering by the Partioning Around Medoids algorithm (PAM) (see the supplemental material) identified 521 transcripts with elevated levels within 9 days after *Hcfc1*^hepKO^ induction (called early upregulated) (Table S3) and 2,871 with elevated levels only at 9 days and beyond *Hcfc1*^hepKO^ induction (called late upregulated) (Fig. 5F and Table S4). Because the latter set was so numerous and late after tamoxifen treatment and, thus, unlikely to represent direct HCF-1 effects, we did not pursue them further here.

To characterize the total downregulated and early upregulated gene sets, we probed the Gene Ontology (GO) database (38) as listed in Tables S5 and S6, respectively. The most significant associated GO term (*P* value of 1.4 × 10^−19^) was for the mitochondrial cellular component term (GO:0005739) with 1,667 genes and the downregulated gene set. One hundred twelve downregulated and 72 upregulated genes were associated with this GO term (Fig. S10). Figure 5B shows REVIGO graphic representations (39), in which the semantic relationship of GO terms with *P* values less than 5 × 10^−4^ were plotted with a *P* value color scale (see Fig. S11 for results for early upregulated genes). Thus, functionally related GO terms neighbor one another on the REVIGO plot.

The GO terms for the *Hcfc1*^hepKO/Y^ downregulated genes included oxidation-reduction, mitochondrial respiratory chain complex I assembly, lipid/fatty acid metabolism, and transport (Fig. 5B and Table S5), all mitochondrial functions. The early upregulated transcripts (Fig. S10B and Table S6) fell into GO terms dominated by immune activation, consistent with the inflammatory response we observe by 7 days (Fig. 3D and Fig. S7), and oxidation-reduction process, again consistent with mitochondrial dysfunction.

To characterize the functions of the *Hcfc1*^hepKO/Y^ downregulated and early upregulated genes further, we performed gene set enrichment analysis (GSEA) with the GSEA Hallmark pathways. In the downregulated set, we identified four pathways: bile-acid metabolism, oxidative phosphorylation, peroxisome, and fatty acid metabolism (Fig. 5C). In contrast, in the early upregulated transcript set, we identified a broad array of pathways, including those for epithelial-to-mesenchymal transition, hypoxia, angiogenesis, apoptosis, tumor necrosis factor alpha signaling, inflammatory response, cell cycle, p53 pathway, MTORC1 signaling, and cholesterol homeostasis (Fig. S11B). Thus, the genes downregulated upon HCF-1 loss, i.e., genes more likely to be activated by HCF-1, form a more coherent set of genes focused on mitochondrial function.

### Multisite genomic localization of HCF-1 in the liver.

To date, chromatin-bound HCF-1 largely has been found located near transcriptional start sites (TSS), with, for example, 743 TSS in proliferating mouse embryonic stem cells (16) and over 5,400 TSS in proliferating human HeLa cells (17). Here, we performed chromatin immunoprecipitation followed by high-throughput sequencing (ChIP-Seq) to examine HCF-1 genomic localization in the liver at days 0 and 4 after induction of hepatocyte-specific *Hcfc1* gene disruption (summarized in Table S7); examples of transcription units with HCF-1 profiles are given in Fig. 5D. After filtering (see Materials and Methods), we identified 7,657 HCF-1 peaks, of which 5,690 peaks (74%) were located within 250 bp up- or downstream of an annotated TSS (Fig. 5E), a number similar to that found in HeLa cells (17). Here, we focused on the 5,690 TSS-associated HCF-1 peaks. All but 63 of the 5,690 TSS-associated HCF-1 peaks disappeared by day 4 (Table S7), as illustrated in Fig. 5D, and nearly all of these remaining peaks reflected residual HCF-1 binding (Fig. S12).

Of the 5,690 TSS-associated HCF-1 peaks, 1,065 (19%) were associated with bidirectional promoters (Fig. 5E) in which the HCF-1 peak was within 250 bp up- or downstream of two TSS. Thus, the 5,690 TSS-associated HCF-1 peaks translate into 6,654 HCF-1-associated TSS (listed in Table S8), of which 33% are associated with bidirectional promoters (Fig. 5E). Thus, HCF-1 displays preferential binding biased for a large number of TSS. Below, we examine the relationship between HCF-1-bound TSS and the gene expression status (i.e., RNA-Seq levels) of the corresponding genes.

### Correspondence between HCF-1-bound TSS and RNA transcript levels.

Indeed, as shown in Fig. 5F (bottom), a high percentage (86%) of HCF-1-associated transcription units had corresponding signals in the RNA-Seq analysis, indicating that HCF-1 largely associates with active transcription units. [Indeed, this percentage of active transcription units is likely greater than 86%, as many of the undetected transcripts corresponded to poly(A)-negative noncoding RNAs which, if synthesized, would be lost owing to our poly(A)-containing transcript selection.] Interestingly, however, most (3,963, or 69%) of the detected HCF-1-associated transcription units did not change measurably as a result of loss of HCF-1. Furthermore, the proportion of down- and early upregulated transcripts (1,175) among the changing transcripts (4,046, or 29%) does not differ much with the same proportion among HCF-1-associated transcription units (443 out of 1,756, or 25%) (Fig. 5F). Thus, remarkably, HCF-1 associates with many transcription units but does not appear to play a determinant role at many of them, and it seems similarly associated with activation or repression of transcription, as indicated previously (17).

### Loss of HCF-1 leads to a corresponding loss of mitochondrial electron transport machinery.

**FIG 6:**
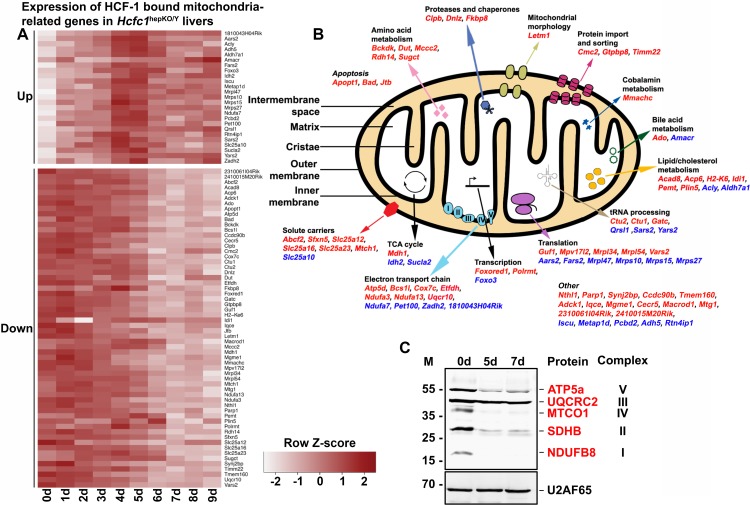
Combined RNA-Seq and ChIP-Seq analyses of control and *Hcfc1*^hepKO/Y^ male livers identifies major alterations in expression levels of HCF-1-bound mitochondrion-related genes upon loss of HCF-1. (A) Heat map of upregulated (top) and downregulated (bottom) mitochondrial-cellular component (GO:0005739) gene-specific RNA levels whose corresponding TSS is bound by HCF-1 from 0 to 9 days after tamoxifen treatment in *Alb-Cre-ERT2*^tg^; *Hcfc1*^hepKO/Y^ males. The color key indicates the associated Z-score. (B) Schematic of a mitochondrion showing functionally annotated genes whose TSS is HCF-1 bound and shows either upregulation (blue) or downregulation (red) upon loss of HCF-1. (C) Immunoblotting with anti-OXPHOS antibody cocktail and anti-U2AF65 loading control antibody with liver lysates of samples collected from control (0d) liver, and *Alb-Cre-ERT2*^tg^; *Hcfc1*^hepKO/Y^ male livers 5 and 7 days after tamoxifen treatment.

In those cases where HCF-1 is TSS associated and the corresponding gene transcripts are down- or upregulated, there is a *prima facie* argument for a direct role of HCF-1 in the regulation of their transcription status. We therefore performed GO analyses on the 280 downregulated and 163 early upregulated genes associated with HCF-1-bound TSS (Fig. 5F). Strikingly, 60 (21%) downregulated and 25 (15%) early upregulated genes belonged to the GO:0005739 mitochondrial cellular component gene set of 1,667 genes, making this gene set the most significant GO cellular component term for both of these two gene sets (Tables S9 and S10). Thus, there is a preponderance of HCF-1 association with genes involved in mitochondrial function. Figure 6A illustrates the behavior of these 60 down- and 25 early upregulated transcripts with a heat map of gene expression changes over 9 days after tamoxifen treatment. Figure 6B shows that these 85 genes are broadly involved in mitochondrial structure and function, consistent with the broad changes observed here in mitochondrial morphology and metabolic activities upon loss of HCF-1.

To probe the effects of loss of HCF-1 directly on oxidative phosphorylation, a key mitochondrial function, we used a five-antibody cocktail (called anti-OXPHOS) that recognizes a single protein subunit within each of the five electron transport complexes (I to V) that is labile upon complex assembly failure. As shown in Fig. 6C, the levels of mitochondrial complex I subunit NDUFB8, complex II subunit SDHB, complex IV catalytic subunit I MTCO1, and complex V subunit ATP5α, but not mitochondrial complex III core protein 2 UQCRC2, were greatly reduced in the *Hcfc1*^hepKO/Y^ males by 5 days after tamoxifen treatment. Consistent with the immunoblot analysis, tissue immunostaining with individual antibodies against ATP5α, MTCO1, and even the marginally reduced UQCRC2 protein showed significant reduction in mitochondrial staining in knockouts compared to levels in control males (Fig. S13A), which was confirmed by a reduced signal with the complete anti-OXPHOS cocktail mix (Fig. S13B). An assay for succinate dehydrogenase (SDH) activity, also a mitochondrial activity, showed reduced activity in *Hcfc1*^hepKO/Y^ males at 7 and 18 days after tamoxifen treatment, where only a few active clusters were seen (Fig. S13C).

Hence, loss of HCF-1 affects expression of genes involved in mitochondrial function and affects the assembly of electron transport chain complexes. The associated mitochondrial dysfunction probably results in inefficient β-oxidation of fatty acids, leading to the observed increased steatosis and rapid progress from NAFLD to NASH.

### Clustered activation of progenitor cell proliferation in knockout males.

Upon liver injury, surviving healthy hepatocytes are known to reenter the cell cycle and to proliferate to restore hepatic function (40–43). However, we have previously shown in a partial-hepatectomy liver regeneration assay that HCF-1 is essential for *de novo* hepatocyte proliferation (22). Hence, upon loss of HCF-1 in the hepatocytes of resting *Hcfc1*^hepKO/Y^ male livers, the pathology may be particularly severe because of not only their dysfunction but also their additional inability to proliferate. Nevertheless, in cases of severe hepatic injury, where healthy hepatocytes are unable to restore hepatic function, alternate pathways involving hepatocyte progenitor cells, generally located near the veins, can be activated (44–48). We therefore investigated here the status of hepatic progenitor cell activation in these knockout male livers in their late phase of disease pathology.

**FIG 7:**
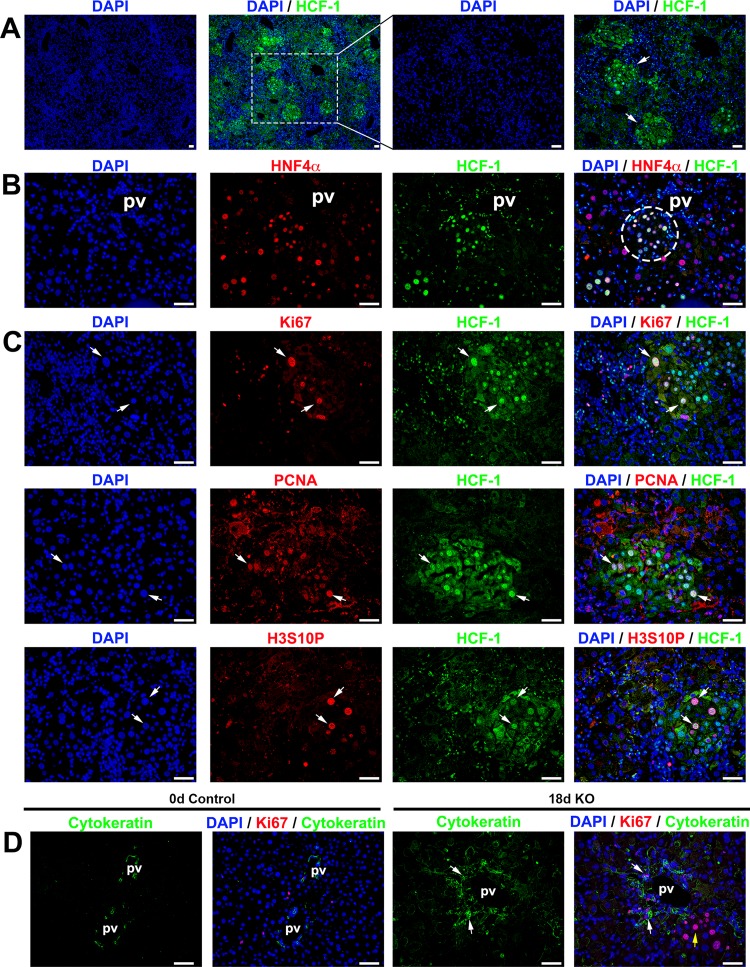
Progenitor cell population is activated in *Hcfc1*^hepKO/Y^ male livers by 18 days after tamoxifen treatment. Immunofluorescence analyses of paraffin-embedded sections from *Alb-Cre-ERT2*^tg^; *Hcfc1*^hepKO/Y^ male livers 18 days after tamoxifen treatment are shown. (A) Staining with DAPI (blue) and HCF-1 antibody (green). Both low and high magnifications of the same section are shown. The arrows point to distinct HCF-1-positive clusters. (B) Staining with DAPI (blue) as well as HNF4α (red) and HCF-1 (green) antibodies. The circle highlights a periportally located HNF4α- and HCF-1-positive hepatocyte cluster. (C) Staining with DAPI (blue) and HCF-1 (green) antibody and, in red, either Ki67 (top), PCNA (middle), or H3S10P (bottom) antibody. Arrows point to Ki67-positive, PCNA-positive, or H3S10P-positve hepatocytes that are also HCF-1 positive. (D) Staining with DAPI (blue) as well as Ki67 (red) and cytokeratin (green) antibodies. White arrows point to periportal Ki67- and cytokeratin-positive cells. The yellow arrow points to a cluster of cells with hepatocyte morphologies. pv, portal vein. Scale bars, 50 μm.

Such an analysis of the *Hcfc1*^hepKO/Y^ knockout male livers 18 days after tamoxifen treatment revealed distinct clusters of HCF-1-positive cells, primarily near hepatic, particularly portal, veins (Fig. 7A) and could be identified even by H&E staining (see the dotted boundary in Fig. S5). Although such HCF-1-positive clusters could arise from HCF-1-positive hepatocytes that escaped *Alb-Cre-ERT2*^tg^-induced deletion of the *Hcfc1*^lox^ allele and, thus, proliferated in response to the surrounding liver injury, it is more likely that they result from activation of a hepatocyte progenitor pathway in part because of their position near hepatic veins where progenitor cells reside. Furthermore, as shown in Fig. 7B, although all the cells in these clusters are positive for the hepatocyte-specific marker HNF4α and, thus, can be defined as hepatocytes, the sizes of their nuclei are heterogeneous, ranging from the small size associated with hepatocyte progenitors to the large size of hepatocytes, suggesting an ongoing evolution from hepatocyte progenitor to fully differentiated hepatocyte.

Indicative of functional hepatocytes, these *de novo* HCF-1-positive hepatocyte clusters displayed low fat content (Fig. 2A, dotted boundary) and higher SDH activity (Fig. S13C, dashed boundary). Many hepatocytes within these clusters were actively proliferating, as they were positive for the cell proliferation marker Ki67, the S-phase marker proliferating cell nuclear antigen (PCNA), and the interphase and mitotic marker histone H3 phosphorylated at serine 10 (H3S10P) (Fig. 7C, arrows).

Cytokeratin is a marker for hepatocyte progenitors (49, 50). Thus, to probe a linkage between the proliferating HCF-1-positive hepatocyte clusters and progenitor cells, we performed immunostaining for cytokeratin. We found that the number of cytokeratin-positive cells was significantly increased near portal vein areas in *Hcfc1*^hepKO/Y^ males and that these cells were actively proliferating, as many were Ki67 positive (Fig. 7D). Thus, the proliferating HCF-1-positive hepatocyte clusters were well positioned to have arisen from hepatocyte progenitor cells. Indeed, Fig. 7D shows a cluster of proliferating hepatocytes adjacent to the cytokeratin-positive cells.

Thus, although there was clear hepatic progenitor cell activation, the response was clearly insufficient to restore hepatic function for survival beyond 21 to 24 days.

### Loss of HCF-1 in clustered hepatocytes leads to transient mild liver injury in *Hcfc1*^hepKO/+^ heterozygous females.

As mentioned above, *Hcfc1*^hepKO/+^ females survive even though, owing to random inactivation of the X-linked *Hcfc1* gene, roughly half the hepatocytes in the liver lose HCF-1 after *Hcfc1*^hepKO^ allele induction (22). Here, we investigated further the impact of this cell-selective loss of HCF-1 on liver function.

**FIG 8:**
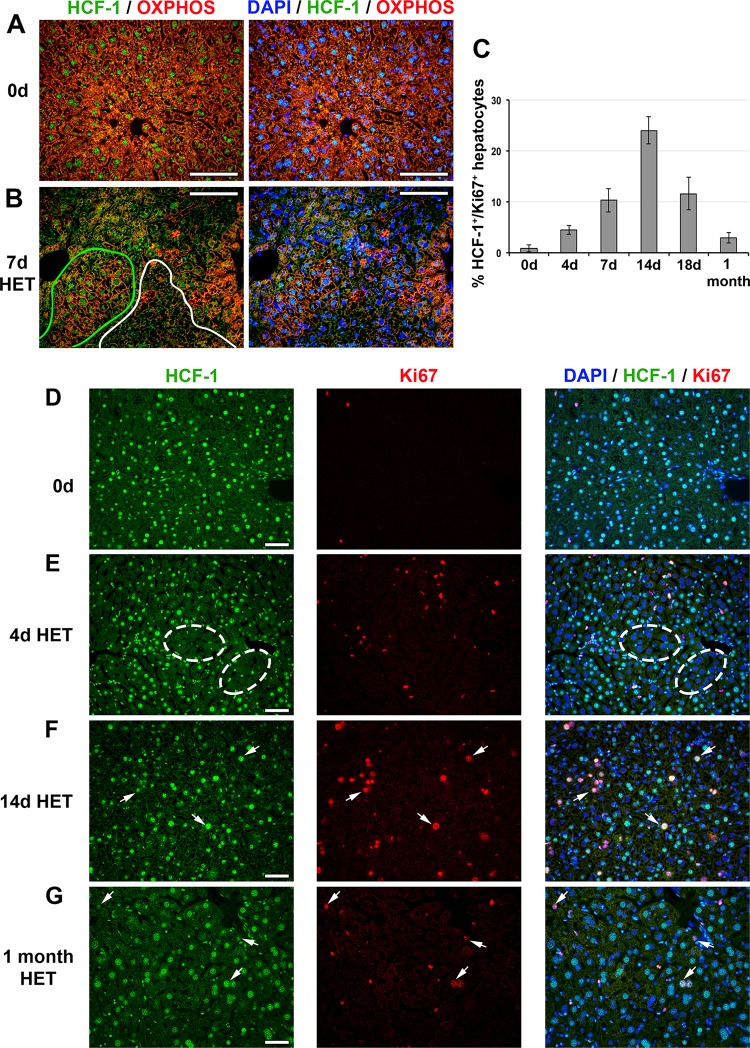
HCF-1-positive hepatocytes proliferate and replace HCF-1-negative hepatocytes in *Hcfc1*^hepKO/+^ heterozygous (HET) females. (A and B) Immunofluorescence analysis of paraffin-embedded sections from control (0d) liver (A) and *Alb-Cre-ERT2*^tg^; *Hcfc1*^hepKO/+^ liver 7 days after tamoxifen treatment (B) stained with DAPI (blue) as well as OXPHOS (red) and HCF-1 (green) antibodies. In panel B, HCF-1-positive (green) and HCF-1-negative (white) clusters are outlined. (C) Percentages of HCF-1-postive-hepatocytes that are Ki67 positive (cycling) in control (*n* = 3), and tamoxifen-treated *Hcfc1*^hepKO/+^ livers after 4 days (*n* = 3), 7 days (*n* = 3), 14 days (*n* = 2), 18 days (*n* = 3), and 1 month (*n* = 2). The differences between percentages of Ki67-positive HCF-1-positive hepatocytes between 0 and 4 days (*P* value of 0.006), 4 and 7 days (*P* value of 0.03), 7 and 14 days (*P* value of 0.03), 14 and 18 days (*P* value of 0.02), and 18 days and 1 month (*P* value of 0.03) after tamoxifen treatment were significant. (D to G) Immunofluorescence of paraffin-embedded sections from control (0d) liver (D) and *Alb-Cre-ERT2*^tg^; *Hcfc1*^hepKO/+^ livers 4 days (E), 14 days (F), and 1 month (G) after tamoxifen treatment stained with DAPI (blue) as well as HCF-1 (green) and Ki67 (red) antibodies. Ovals point to clusters of HCF-1-negative hepatocytes. Arrows point to HCF-1-positive, Ki67-positive hepatocytes. Scale bars, 100 μm.

Consistent with the loss of HCF-1 within 4 days of *Hcfc1*^hepKO^ allele induction in males, in *Hcfc1*^hepKO/+^ females, patches of HCF-1-positive and -negative hepatocytes could be observed by 4 days, and as in knockout males, the nonhepatocyte liver cell types remained HCF-1 positive (Fig. 8E, ovals). In contrast to the severe liver pathology of male *Hcfc1*^hepKO/Y^ mice at 18 days, however, H&E (Fig. S14A) and β-catenin immunostaining (Fig. S14B and C) of the *Hcfc1*^hepKO/+^ female livers showed only mild long-term tissue disorganization. Furthermore, liver functions were only transiently affected. Thus, an increase in hepatic fat content at 7 days was rapidly resolved by 11 days after *Hcfc1*^hepKO^ allele induction (Fig. S15A), and periodic acid-Schiff staining showed a slight decline in glycogen stores at 11 days, with near-normal levels observed by 18 days after *Hcfc1*^hepKO^ allele induction (Fig. S14D and S15B).

As the AST liver injury marker levels were marginally increased in *Hcfc1*^hepKO/+^ females (Fig. 1D), we investigated inflammation and liver injury markers. Indeed, in *Hcfc1*^hepKO/+^ females, there was an increase in F4/80-positive macrophages from 9 days after *Hcfc1*^hepKO^ allele induction (Fig. S14E and S16), indicating an inflammatory and eventually regenerative response to the presence of HCF-1-negative cells. Albeit an evident inflammatory response, the effect on the liver structure was relatively mild, as there was little if any activation of SMA-positive stellate cells (Fig. S14F) or deposition of Sirius red-positive collagen fibers (Fig. S14G and S17). These results indicate that *Hcfc1*^hepKO^ allele induction in heterozygous females leads to a transient liver pathology with little lasting structural damage.

This transient liver pathology likely owes to the loss of HCF-1 function in roughly half the hepatocytes of *Hcfc1*^hepKO/+^ females. For example, coimmunostaining with anti-OXPHOS and anti-HCF-1 showed decreased levels of OXPHOS-positive cells only in HCF-1-negative cell clusters compared to levels in HCF-1-positive cell clusters (Fig. 8A and B, cluster outlines). Thus, importantly, in a side-by-side comparison, the loss of mitochondrial function is directly linked to the loss of HCF-1, supporting a cell-autonomous role of HCF-1 in mitochondrial function.

Congruous with resolution of the liver pathology after *Hcfc1*^hepKO^ allele induction in the heterozygous females, by 1 month after *Hcfc1*^hepKO^ allele induction, HCF-1-positive hepatocytes were replacing HCF-1-negative hepatocytes (Fig. 8G). Indeed, HCF-1-positive hepatocytes reentered the cell cycle, as evidenced by anti-Ki67 labeling of HCF-1-positive cells (Fig. 8E to G and Fig. S18). As quantified in Fig. 8C, the percentage of HCF-1/Ki67 double-positive hepatocytes increased over time after *Hcfc1*^hepKO^ allele induction, with a maximum at 14 days. Furthermore, in parallel, cell death detected by TUNEL staining appeared clustered in HCF-1-negative cells in the *Hcfc1*^hepKO/+^ livers at 18 days (Fig. S14H).

In summary, *Hcfc1*^hepKO/+^ females experience liver injury, similar to initial stages of NAFLD, upon clustered cell loss of HCF-1. They survive apparently owing to replacement of dysfunctional HCF-1-negative hepatocytes removed by apoptosis with functional HCF-1-positive cells created by cell cycle reentry and subsequent proliferation. Hence, the heterozygote females represent a model for resolution of NAFLD.

## DISCUSSION

Experimental studies of NAFLD have utilized animal models in which disease onset is induced by either high-fat diets (for example, high-fat diet lacking methionine and choline, essential for beta-oxidation and very-low-density lipoprotein secretion), with or without toxic chemical insult (e.g., carbon tetrachloride), or by using genetic strains where animals accumulate excess fat, usually in combination with high-fat diets (12–15, 51). Although all these models show fat accumulation, not all progress to advanced stages of NAFLD, and others that do take several months to induce successfully. Hence, the rapid 3-week progression to advanced NAFLD described here with *Hcfc1*^hepKO/Y^ males and pathology resolution observed with *Hcfc1*^hepKO/+^ females should, in combination, be useful alternative models for NAFLD.

The hepatocyte-specific *Hcfc1* knockout in *Hcfc1*^hepKO/Y^ males models the development of terminal NASH characterized by increased steatosis, mitochondrial defects, increased inflammation and cell death, hepatocyte ballooning, fibrosis, progenitor cell activation, and metabolic dysfunction. In the absence of functional hepatocytes, progenitor cells near hepatic veins proliferate, forming small areas of newly formed hepatocytes characterized by the presence of increased proliferation markers, decreased lipid content, and normal mitochondrial activity (Fig. 7). Although, by their location near hepatic veins, these progenitors may well represent hybrid periportal hepatocytes (45), we have not made a direct determination here. The newly generated hepatocytes, however, fail to restore full liver function, and the mice succumb to terminal NASH. These *Hcfc1*^hepKO/Y^ mice represent a new genetic model of NASH that is rapidly induced without the use of any dietary supplements.

The hepatocyte-specific heterozygous *Hcfc1* knockout in *Hcfc1*^hepKO/+^ females models rapid resolution of liver injury characterized by steatosis and fibrosis as well as inflammation. Remaining HCF-1-positive cells rapidly replace the injured HCF-1-negative cells, and livers revert to normal architecture and function within about a month. In addition to permitting the study of a resolution response, these mice offer the added advantage of the possibility of studying proliferative (in HCF-1-positive patches) and apoptotic (in HCF-1-negative patches) hepatocytes within the same overall liver environment. Such a genetically induced model of NAFLD displaying rapid resolution is not currently available.

Soon after induction of complete hepatocyte-specific loss of HCF-1 in *Hcfc1*^hepKO/Y^ males, there is increased steatosis accompanied by an alteration in mitochondrion-related gene expression levels, which is soon followed by metabolic dysfunction. Genes downregulated upon loss of HCF-1 are most significantly involved in mitochondrial structure and function, including oxidation-reduction, whereas the genes that are upregulated upon loss of HCF-1 are involved in stress responses, such as innate immune response seen upon injury. Dysfunctional mitochondrial activities are known to play a key role in the pathogenesis of NAFLD and the transition to NASH. Hence, our results suggest that a primary reason for rapid progression of *Hcfc1*^hepKO/Y^ male mice to advanced NAFLD stages is mitochondrial dysfunction.

### HCF-1 roles in hepatocyte function.

HCF-1 likely plays multiple roles in liver physiology via at least two avenues: the stabilization of PGC1α protein and regulation of gene transcription.

Probably one reason *Hcfc1*^hepKO/Y^ male mice progress rapidly through NAFL to terminal NASH owes to the destabilization of PGC1α protein by the absence of HCF-1, as shown here and by Ruan et al. (28). Indeed, there are many similarities between PGC1α knockout mice and *Hcfc1*^hepKO/Y^ male mice, including impairment of gluconeogenesis, increased fat accumulation, downregulation of genes involved in fatty acid oxidation, and increased inflammation (37, 52, 53). However, the PGC1α knockout mice are not terminally ill and do not progress to NASH, whereas *Hcfc1*^hepKO/Y^ male mice do. Thus, inactivation of a second role of HCF-1 is likely to be responsible for the more severe phenotype associated with loss of HCF-1.

Loss of HCF-1 leads to a gross disruption of gene expression, as evidenced by the marked changes in gene transcript levels soon after induction of HCF-1 loss. Furthermore, the ChIP-seq results show that HCF-1 is principally associated with the TSS of transcriptionally active genes, as evidenced by the detection of corresponding gene transcripts. Thus, we suggest that a second important function of HCF-1 in hepatocytes is in transcriptional regulation.

Nevertheless, this role is curious. HCF-1 is bound to a very large number of active TSS, 5,719 detected here, yet the levels of transcript activity of less than one-third of these HCF-1-bound TSS (31%) change upon loss of HCF-1. Thus, there is clearly no apparent absolute requirement for HCF-1 to maintain activated or repressed levels of gene transcription of most HCF-1-associated transcription units. Rather, it may be commonly associated with transcriptional regulatory promoter complexes but only directly influence transcriptional regulation of a subset of these. In the context of hepatocytes, however, this subset is enriched in genes involved in a wide variety of mitochondrion-associated functions, which apparently leads to rapid liver dysfunction upon its disappearance.

Whichever the case, after long being known for its role in cell proliferation (19), this study illustrates the life-sustaining role HCF-1 can play in the functions of a differentiated tissue, the resting adult liver. Thus, HCF-1 critically potentiates the cellular functions of both proliferating and nonproliferating cells through a multiplicity of mechanisms yet to be fully elucidated.

## MATERIALS AND METHODS

### Mice.

All experimental studies have been performed in compliance with EU and national legislation rules, as advised by the Lemanique Animal Facility Network (Resal), concerning ethical considerations of transportation, housing, strain maintenance, breeding, and experimental use of animals.

Homozygous mice bearing the *Hcfc1* conditional (lox) allele are referred to as *Hcfc1*^lox/lox^ (22). The *Hcfc1*^lox^ allele becomes the *Hcfc1*^cKO^ allele in the presence of Cre recombinase, encoding a highly truncated 66-amino-acid-long HCF-1 protein (22).

Other strains used in this study include wild-type C57BL/6 mice and *Alb-Cre-ERT2*^tg^ transgenic mice (54) (a gift from Daniel Metzger, IGBMC Strasbourg).

Females and littermate males were housed four or five per cage at 23°C, with a 12-h/12-h light/dark cycle and *ad libitum* access to water and food. Tamoxifen treatment and all tissue collections were performed at ZT2 to -3, where ZT0 is the start of the light period.

### DNA isolation and genotyping.

For genotyping, genomic DNA was isolated from postnatal mouse ear tags as previously described (55). Samples were used for PCR amplification with specific primer sets using the KAPA2G Fast HotStart genotyping PCR mix (no. KK5621).

### Tamoxifen induction.

Control female *Hcfc1*^lox/+^ and male *Hcfc1*^lox/Y^ mice and test female *Alb-Cre-ERT2*^tg^; *Hcfc1*^lox/+^ and male *Alb-Cre-ERT2*^tg^; *Hcfc1*^lox/Y^ mice (10 to 14 weeks old) were administered intraperitoneal injections of 1 mg/mouse tamoxifen (100 μl of 10 mg/ml [1:10 ethanol-corn oil]; 10540-29-1; Sigma-Aldrich) three times at 24-h intervals from day 0 to day 2.

### Tissue histology and immunohistochemistry.

For fluorescence and diaminobenzidine (DAB) immunostaining and colorations (H&E, PAS, and Sirius red stainings and TUNEL assay), the liver tissues were sectioned into 4-μm-thick paraffin sections. For immunostaining, sections were incubated overnight with the respective primary antibodies. For Oil Red O staining and succinate dehydrogenase assay, the liver tissues were sectioned into 8-μm-thick cryosections. Additional details are provided in the supplemental material.

### Immunoblotting.

Freshly isolated liver tissue (100 mg) was homogenized in radioimmunoprecipitation assay buffer and protein concentrations were determined. Additional details are provided in the supplemental material.

### Quantitation and statistical analyses.

For each analysis, all cells in a representative field of 4-µm-thick paraffin sections were counted. Student’s *t* tests were performed by using the R package (www.r-project.org).

### Data availability.

We declare that the data supporting the findings of this study are available within the paper and in the supplemental material.

## Supplementary Material

Supplemental file 1

Supplemental file 2

Supplemental file 3
