# Supplementary material for: Rapid Recapitulation of Nonalcoholic Steatohepatitis upon Loss of Host Cell Factor 1 Function in Mouse Hepatocytes
Source: Mol Cell Biol. 2019 Feb 15;39(5):e00405-18. doi: 10.1128/MCB.00405-18 (PMC6379584; doi:10.1128/MCB.00405-18)
Supplement: Supplemental file 1 [file d2332be1aec526d17a250b18cae15be9_MCB.00405-18-s0001.pdf]

Supporting Information

For

Rapid recapitulation of non-alcoholic steatohepatitis upon loss of HCF-1 function in mice

Shilpi Minocha[1], Dominic Villeneuve[1], Viviane Praz[1,2], Catherine Moret[1], Maykel Lopes[1], Danièle Pinatel[1], Leonor Rib[1,2,+], Nicolas Guex[2], and Winship Herr[1,*]

Email of authors:

Shilpi Minocha: shilpi.minocha@unil.ch,

Dominic Villeneuve: dominic.s.villeneuve@gmail.com,

Viviane Praz: viviane.praz@unil.ch,

Catherine Moret: catherine.moret@unil.ch,

Maykel Lopes: maykel.lopes@unil.ch

Danièle Pinatel: daniele.pinatel@unil.ch

Leonor Rib: leonor.rib@unil.ch

Nicolas Guex: nicolas.guex@unil.ch

Winship Herr: winship.herr@unil.ch

Institutional affiliation of authors:

[1] Center for Integrative Genomics, Génopode, University of Lausanne, CH-1015 Lausanne, Switzerland.

[2] Swiss Institute of Bioinformatics, Génopode, University of Lausanne, 1015 Lausanne, Switzerland.

+ Present address: The Bioinformatics Center, Department of Biology & Biotech Research and Innovation Center, University of Copenhagen, 2200 Copenhagen, Denmark.

* **Corresponding author**: Winship Herr, Center for Integrative Genomics, Génopode, University of Lausanne, 1015 Lausanne, Switzerland. Tel: +41 21 692 3922; email: winship.herr@unil.ch.

Supplementary experimental procedures

Primers and PCR conditions for genotyping:

For HCF-1: p1 (5'-GGAGGAACATGAGCTTTAGG-3'), p2 (5'-CAATAGGCGAGTACCATCACAC-3'), and p3 (5'-GGGAAAGTAGACCCACTCTG-3'). The annealing was done at 62°C for 15 seconds with an extension at 72°C for 10 seconds (22).

For AlbCre: p1 (5'-ATCATTTCTTTGTTTTCAGG-3'), p2 (5'-GGAACCCAAACTGATGACCA-3'), and p3 (5'-TTAAACAAGCAAAACCAAAT-3'). The annealing was done at 53°C for 1 minute with an extension at 72°C for 1 minute. Combination of p1 and p2 was used to detect the *wildtype* allele (229bp). Combination of p2 and p3 was used to detect the *Cre* allele (444bp).

***Tissue immunohistochemistry and histology*:**

For (a) fluorescence and diaminobenzidine (DAB) immunostaining, and (b) colorations, the liver tissues were either paraffin-embedded and sectioned into 4 µm thick sections using a MICROM HM325 microtome or cut with the help of a cryostat MICROM HM550 microtome to generate 8 µm thick sections.

(a) *Fluorescence and DAB immunostaining*: The paraffin-embedded sections were first (i) deparaffinized in xylene, (ii) rehydrated through graded alcohol washes, and (iii) rinsed twice with PBS. For DAB immunostaining, endogenous peroxidase activity was quenched at this stage with 6% hydrogen peroxide in methanol for 10 min and rapidly washed once with H_2O. Subsequently antigens for both fluorescence and DAB immunostaining were revealed by heating in a 750 W microwave oven until boiling for approximately 10 min in citrate buffer (10mM, pH 6.0), allowed to slowly cool to 4°C, washed twice with PBS, and then blocked for 30 min with 2% normal goat serum (NGS)

(Sigma-Aldrich, cat. # G9023) in PBS at room temperature (RT). After blocking, primary immunostaining was performed by incubation of the slices with specific primary antibody (see below) diluted in 2% NGS overnight at 4°C followed by three washes with PBS.

For secondary fluorescence immunostaining, incubation with the appropriate secondary antibody (see below) was for 30 min in the dark at RT, followed by (i) three PBS washes, (ii) counterstaining with 4',6-diamidino-2-phenylindole (DAPI) (Sigma-Aldrich, CAS # 28718-90-3), (iii) two PBS washes, and (iv) embedding with Mowiol mounting medium (Sigma-Aldrich, CAS # 9002-89-5). The sections were subsequently analyzed using an AxioImager M1 microscope with AxioCam MRm monochrome and AxioCam MRc color cameras (Carl Zeiss AG, Oberkochen, Germany), or a Zeiss CLSM 710 spectral confocal laser scanning microscope. Images were processed using AxioVision 4.8.2 (Carl Zeiss AG, Oberkochen, Germany) or Imaris 8.2 (Bitplane Inc.) software.

For secondary DAB immunostaining, the primary antibodies were detected by incubating the sections for 30 min with anti-mouse (Dako cat. # K4000) or anti-rabbit (Dako cat. # K4002) horseradish peroxidase (HRP) secondary antibody. Visualization was performed with DAB substrate (Dako cat. # K3468) before being counterstained with Mayer’s hematoxylin. The sections were analyzed using the AxioImager M1 microscope with AxioCam MRm monochrome and AxioCam MRc color cameras (Carl Zeiss AG, Oberkochen, Germany). Tilescan imaging of entire liver lobes was acquired at high resolution using a Leica DMi8 inverted microscope with Andor Zyla black and white and DFC7000T color cameras.

The primary antibodies used were: rabbit anti-HCF-1 (1:1000, H12, (56)), rat anti-Ki67 (1:60, eBioscience cat. # 41-5698), anti-HNF4α (1:100, R&D Systems cat. #

PP-H1415-00), anti-β-catenin (1:75, BD Biosciences, cat. # 610153), anti-SMA (1:400, Abcam cat. # ab5694), anti-Oxphos (1:100, Abcam cat. # ab110413), anti-PGC1α (1:100, Millipore cat. # ST1202), anti-ATP5a (1:400, Abcam cat. # ab14748), anti-UQCRC2 (1:400, Abcam cat. # ab14745), anti-MTCO1 (1:200, Abcam cat. # ab14705), and anti-PCNA (1:50, BD Transduction Laboratories cat. # 610665) mouse monoclonal antibodies, rabbit anti-Histone H3 phospho Ser10 (1:100, Abcam cat. ab5176), rabbit anti-cytokeratin (1:100, Dako cat. # Z0622), rat anti-F4/80 (1:800, Abcam cat. # ab6640), and rat anti-CD31 (1:20, Dianova GmbH cat. # DIA-310-M).

The secondary antibodies used were: goat anti-rabbit Alexa 488 (1:400, Molecular Probes cat. # A11034), goat anti-mouse Alexa 568 (1:500, Molecular Probes cat. # A11019), goat anti-rabbit Alexa 568 (1:1000, Molecular Probes cat. # A21069), goat anti-mouse Alexa 488 (1:400, Molecular Probes cat. # A11029), donkey anti-mouse Alexa 594 (1:500, Molecular Probes cat. # A11005), and goat anti-mouse Alexa 635 (1:300, Molecular Probes cat. # A31575).

b) Colorations:

Hematoxylin (H) and Eosin (E) staining

Standard hematoxylin and eosin staining was performed on deparaffinized and rehydrated liver sections (57).

Periodic acid-Schiff (PAS) staining

For glycogen detection, PAS staining was performed on deparaffinized and rehydrated liver sections by incubation in 1% periodic acid for 10 mins followed by staining with Schiff's reagent for 15 mins, followed by Mayer’s hematoxylin staining for 5 mins.

Sirius red staining

Sirius red staining was done to assess collagen deposition in injured liver paraffin sections. After hydrating, sections were rinsed in 0.2% phosphomolybdic acid for 5 minutes followed by staining with 0.1% sirius red stain for 90 minutes. Thereafter, the sections were stained with Mayer's hematoxylin for 1 minute.

Oil Red O assay

Liver samples were cut with the help of a cryostat microtome (MICROM HM550) to generate 8 μm thick cryo-sections for this assay. After drying the cryo-sections were (i) fixed with 4% paraformaldehyde (PFA) for 10 minutes at RT, (ii) quickly washed with distilled water, (iii) rinsed with 60% isopropanol for 1 minute, (iv) stained with Oil Red O solution (Sigma cat. # 0625) for 10 minutes, (v) quickly rinsed with 60% isopropanol, (vi) quickly rinsed with distilled water, (vii) co-stained with hematoxylin for 45 seconds, (viii) washed very well with water, and (ix) mounted. With this staining protocol, lipids and nuclei can be seen in red and blue color, respectively.

Succinate dehydrogenase assay

Liver samples were cut with the help of a cryostat microtome (MICROM HM550) to generate 8 μm thick cryo-sections for this assay. After drying, the cryo-sections were incubated in succinate dehydrogenase solution containing succinic acid (Sigma cat. # S2378) and nitro blue tetrazolium chloride, NBT (Roche cat. # 11 383 213 001) for 30 minutes at 37°C. Thereafter, the sections were washed thrice with 1X PBS. This staining causes formation of a purple precipitate over top of mitochondria.

***Immunoblotting*:**

For immunoblotting, approximately 100 mg of liver tissue from each time point was homogenized in RIPA buffer (50 mM Tris-HCl ph7.4, 150 mM NaCl, 1 mM EDTA, 0.2% sodium deoxycholate, 1 mM DTT, 1mM PMSF, and 1% Triton X) containing protease inhibitor (Roche). Samples (10-20 μg) were boiled for 5 mins before PAGE and transfer to nitrocellulose membrane. Membranes were blocked for 60 mins with 5 ml of LI-COR blocking buffer, incubated with primary antibody in 50% LI-COR blocking buffer and 50% PBST (PBS containing 0.1% Tween 20) overnight at 4°C, washed three times and incubated with secondary antibody (dilution 1:10,000) for 30 mins at RT. The membranes were washed three times and scanned with an Odyssey infrared imager (LI-COR).

***TUNEL assay*:**

Terminal deoxynucleotidyltransferase-mediated dUTP-biotin nick end labeling (TUNEL) was performed on paraffin-embedded liver sections with the *in situ* cell death detection kit (Roche Applied Science, cat. # 11684795910), according to the manufacturer's directions.

***Metabolic Assessments*:**

Glucose and pyruvate tolerance tests: After a 15-hour overnight fast, age- and sex-matched 12– to 14–week old mice received glucose or pyruvate by intraperitoneal injections (2 g/kg, as a 20% solution). Blood samples were obtained from tail-tip bleedings, and blood glucose levels were measured with a Glucometer (Glucotrend Premium; Boehringer Mannheim GmbH, Mannheim, Germany). Glycemic areas were measured from time 0 to 120 minutes.

Insulin tolerance tests: After a 5-hour fast, age- and sex-matched 10– to 16–week old mice received insulin by intraperitoneal injections (0.3U/kg). Blood samples were collected like glucose/pyruvate tolerance tests. Plasma insulin levels were determined by ELISA

(Mercodia AB, Uppsala, Sweden).

Diagnostics: Aspartate transaminase (AST) levels were measured by using the Cobas C111 robot (Roche Diagnostics). Alanine transaminase (ALT), HDL/LDL/total cholesterol and triglyceride levels were measured by using the Xpand clinical chemistry system (Siemens Healthcare GmbH).

Estimation of metabolites, energy metabolism intermediates, and bile acids: All these measurements were done at a metabolomics facility (Biocrates Life Sciences AG, Austria). To extract metabolites, age- and sex-matched 10– to 14–week old mice liver samples were homogenized using Precellys® with ethanol phosphate buffer. An aliquot of each sample was centrifuged and the supernatant was used for analysis. AbsoluteIDQ® p180 kit assay was used for the quantification of amino acids, acylcarnitines, sphingomyelins, phosphatidylcholines, hexoses, and biogenic amines. The fully automated assay was based on PITC (phenylisothiocyanate)-derivatization in the presence of internal standards followed by FIA-MS/MS (acylcarnitines, lipids, and hexose) and LC/MS (amino acids, biogenic amines) using an AB SCIEX 4000 QTrap® mass spectrometer (AB SCIEX, Darmstadt, Germany) with electrospray ionization.

For the quantitative analysis of energy metabolism intermediates (glycolysis, citrate cycle, pentose phosphate pathway, urea cycle) hydrophilic interaction liquid chromatography (HILIC)-ESI-MS/MS method in highly selective negative MRM detection mode was used. The MRM detection was performed using an ABSCIEX 4000 QTrap® tandem mass spectrometry instrument (AB SCIEX, Darmstadt, Germany). The sample was protein precipitated and extracted simultaneously with aqueous methanol in a 96 well plate format. Internal standards (ratio external to internal standard) and external calibration were used for highly accurate

quantitation.

A highly selective reversed phase LC-MS/MS analysis method in negative MRM detection mode was applied to determine the concentration of bile acids. Samples were extracted via dried filter spot technique in 96 well plate format, which is well suitable for high throughput analysis. For highly accurate quantification internal standards and external calibration were applied. In brief, internal standards and 10 µL sample volume placed onto filter spots were extracted and simultaneously protein precipitated with aqueous methanol. These sample extracts were measured by LC-ESI-MS/MS with a tandem mass spectrometry instrument (AB SCIEX, Thermo Scientific, Waters). Data of bile acids were quantified with a corresponding MS software (AB SCIEX – Analyst, Thermo Scientific – x-calibur, Waters - Masslynx, Darmstadt, Germany) and finally exported into MetIDQ software for comprehensive statistical analysis.

RNA-sequencing **(*RNA-seq*)**:

Poly(A)-containing RNA from individual livers was used for RNA-seq. RNA was extracted with RNeasy kit (Qiagen cat. # 74104). The disruption and homogenization was done with TissueLyser II. Samples were quantified with MySpec machine and 200µg (in 100µl) of RNA sample was used for further analysis. Strand-specific libraries were prepared with the TruSeq Stranded mRNA Library Prep kit (Illumina catalog # RS-122-9004DOC). The fragment ends (50 nucleotides) were sequenced with single-end sequencing technology from HiSeq 2100 (Illumina).

RNA-seq reads were aligned to the Mouse mm9 genome assembly and transcriptome using TopHat version 2.0.13 with Bowtie version 1.1.2. Counts per gene were calculated using the Rsubreads R package and transformed in RPKM. Normalization was done via the edgeR R package using TMM. The cutoff for genes considered as non-transcribed was calculated

according to the method described in https://bmcgenomics.biomedcentral.com/articles/10.1186/1471-2164-14-778. Genes showing a change during the time-course between zero and ten days were selected using MaSigPro with a significance level of 0.05. The 4549 selected genes were then submitted to a PAM analysis and split into three categories, namely: 1) genes going down during time-course, 2) genes going up during the first half of the time-course, and 3) genes going up during the second half of the time-course. The mean value, and the fold-change (KO minus WT) were calculated for each time point. The selected genes were ranked by the fold-change, and a GSEA was performed using this ranked gene list against the HALLMARKS set (http://software.broadinstitute.org/gsea/msigdb/collection_details.jsp#H).

***ChIP-seq* analysis**:

Chromatin isolation, immunoprecipitation, and preparation of high throughput sequencing: Chromatin was isolated from the liver of individual male *Alb-Cre-ERT2*^{tg} ; *Hcfc1*$^{lox/Y}$ mice at 0 days and at 4 days after *Hcfc1*^{hepKO}-allele induction by tamoxifen administration as described in Methods. Chromatin was immunoprecipitated and prepared for high-throughput sequencing as described (58) using the anti-HCF-1 H12 antibody (56).

Mapping: Both sequenced fragment ends R1 and R2 were first mapped individually on the Mouse genome Mm10 release, allowing up to 50 multiple matches. For both ends, the best matches were extracted for each read. All multiple matches with the same mapping scores were kept at this stage. The two mapped reads R1 and R2 were then merged. If whole fragments had multiple matches with the same score, only the matches giving fragments smaller than 1 kb were kept. The multiple matches were weighted according to the ratio (times

sequenced)/(number of multiple matches). Regions from the ENCODE blacklisted list as well as telomeric regions were discarded for further analysis.

Peak detection: Peaks were first detected with MACS2, with an FDR of 0.01. Those peaks were then compared to the enriched regions determined by another method using genomic bins, as described in Renaud et al (Genome Res. 2014 Jan;24(1):37-51. doi: 10.1101/gr.161570.113. Epub 2013 Oct 9, PMID: 24107381). Only the MACS peaks overlapping with the enriched genomic bins were kept. Peaks positions were then compared sequentially to the following annotated features:

- Pol2 TSS from the Gencode genomic annotation, Mouse Mm10
- Repeats (Alus, MIR, LTR, satellite regions)
- RefSeq genes, coding and non-coding
- Ensembl genes

Scoring: Fragments within a window of minus 250 to plus 250 nucleotides around the Pol2 TSSs (Gencode annotation, Mouse Mm10) were summed, in the IP and in the corresponding Input. One pseudocount was added for log2 calculation. The final score was then calculated as follows: score=log2(IP)-log(Input). All scores were scaled to a total of 15 mio fragments per experiment.

Electron microscopy:

Small pieces of liver (2 mm^3) were fixed in 2.5% glutaraldehyde solution (EMS, Hatfield, PA, USA) in phosphate buffer (PB 0.1 M, pH 7.4) (Sigma, St Louis, MO, USA) for 2 hours at room temperature (RT). Then, the samples were post-fixed in 1% osmium tetroxide (EMS, Hatfield, PA, USA) in phosphate buffer (PB 0.1 M, pH 7.4) (Sigma, St Louis, MO, USA) for 2 hours at

RT. The samples were then washed two times in distilled water and dehydrated in acetone solution (Sigma, St Louis, MO, USA) at graded concentrations (30%-30 min; 70%-30 min; 100%-2x1 h). This procedure was followed by infiltration in epoxy resin (Sigma, St Louis, MO, USA) at graded concentrations (Epon 1/3 acetone-1 h; Epon 3/1 acetone-1 h, Epon 1/1-2 h, Epon 1/1-12h). Pieces of liver were then placed in molds filled with resin and then polymerized for 48 h at 60°C in an oven. Ultrathin sections (50 nm) were cut on Leica Ultracut (Leica Mikrosysteme GmbH, Vienna, Austria) and picked up on a copper slot grid 2x1 mm (EMS, Hatfield, PA, USA) coated with a polystyrene film (Sigma, St Louis, MO, USA). Micrographs were taken with a Philips CM100 TEM (FEI, Eindhoven, The Netherlands) at an acceleration voltage of 80 kV with a TVIPS TemCam-F416 digital camera (TVIPS GmbH, Gauting, Germany).

Supplementary Figure Legends

Supplementary Figure 1. Knockout *Alb-Cre-ERT2*^{tg} ; *Hcfc1*$^{\text{hepKO/Y}}$ males display rapid loss of *Hcfc1* expression whereas heterozygous *Alb-Cre-ERT2*^{tg} ; *Hcfc1*$^{\text{hepKO/Y}}$ females continue to maintain *Hcfc1* expression. View of the reads mapped on the 26 exons of the *Hcfc1* gene, shown in blue at the top, in control *Hcfc1*$^{\text{lox/Y}}$ male (A), heterozygous *Alb-Cre-ERT2*^{tg} ; *Hcfc1*$^{\text{hepKO/+}}$ female (B), and knockout *Alb-Cre-ERT2*^{tg} ; *Hcfc1*$^{\text{hepKO/Y}}$ male (C) livers beginning from 0d to 14d post-tamoxifen treatment with RNA-seq analysis. Arrows point to the reads observed at exon 2 and 3 of the *Hcfc1* gene. The total *Hcfc1* transcript expression level in control *Hcfc1*$^{\text{lox/Y}}$ male (blue), heterozygous *Alb-Cre-ERT2*^{tg} ; *Hcfc1*$^{\text{hepKO/+}}$ female (green), and knockout *Alb-Cre-ERT2*^{tg} ; *Hcfc1*$^{\text{hepKO/Y}}$ male (red) livers is depicted as log2(Reads Per Kilobase of transcript per Million mapped reads or RPKM) values.

Supplementary Figure 2. *Hcfc1*$^{\text{hepKO/+}}$ heterozygous female livers do not show any abnormal features. (A) Macroscopic comparison of livers from control liver (0d), and 7d, 14d, and 18d post-tamoxifen treated heterozygous *Alb-Cre-ERT2*^{tg} ; *Hcfc1*$^{\text{hepKO/+}}$ female livers. (B) Boxplot showing liver-to-body weight ratio of control liver (0d; n=9), and 7d (n=4) and 18d (n=4) post-tamoxifen treated knockout *Alb-Cre-ERT2*^{tg} ; *Hcfc1*$^{\text{hepKO/Y}}$ male livers. The difference between liver-to-body weight ratio of 0d control liver and 7d post-tamoxifen treated knockout *Alb-Cre-ERT2*^{tg} ; *Hcfc1*$^{\text{hepKO/Y}}$ male liver was significant (p-value 0.01). The difference between liver-to-body weight ratio of 7d and 18d post-tamoxifen treated knockout *Alb-Cre-ERT2*^{tg} ; *Hcfc1*$^{\text{hepKO/Y}}$ male livers was marginally significant (p-value 0.05). The difference between liver-to-body weight ratio of 0d control liver and 18d post-tamoxifen treated knockout *Alb-Cre-ERT2*^{tg} ; *Hcfc1*$^{\text{hepKO/Y}}$ male liver was significant (p-value 0.03). (C) Boxplot showing liver-to-body weight ratio of control liver (0d; n=4), and 7d (n=4) and 18d (n=2) post-tamoxifen treated heterozygous *Alb-Cre-ERT2*^{tg} ; *Hcfc1*$^{\text{hepKO/+}}$ female livers. Scale bar: 1 cm.

Supplementary Figure 3. *Hcfc1*$^{\text{hepKO/Y}}$ knockout males display metabolic abnormalities. (A) Graphs showing the body weights of control *Hcfc1*$^{\text{lox/Y}}$ (n=7/time point) and knockout *Alb-Cre-ERT2*^{tg} ; *Hcfc1*$^{\text{hepKO/Y}}$ (n=11/time point) males after tamoxifen treatment. (B) Glucose levels (or glycemia) during glucose tolerance test in control *Hcfc1*$^{\text{lox/Y}}$ (n=6) and knockout *Alb-Cre-ERT2*^{tg} ; *Hcfc1*$^{\text{hepKO/Y}}$ (n=6) males 7d post-tamoxifen treatment. (C) Glycemia during glucose tolerance test in control *Hcfc1*$^{\text{lox/+}}$ (n=5) and heterozygous *Alb-Cre-ERT2*^{tg} ; *Hcfc1*$^{\text{hepKO/+}}$ (n=6) females 7d post-tamoxifen treatment. (D) Glycemia during pyruvate tolerance test in control *Hcfc1*$^{\text{lox/Y}}$ (n=6) and knockout *Alb-Cre-ERT2*^{tg} ; *Hcfc1*$^{\text{hepKO/Y}}$ (n=8) males 4d post-tamoxifen treatment. (E) Glycemia during pyruvate tolerance test in control *Hcfc1*$^{\text{lox/+}}$ (n=5) and heterozygous *Alb-Cre-ERT2*^{tg} ; *Hcfc1*$^{\text{hepKO/+}}$ (n=6) females 4d post-tamoxifen treatment. (E) Basal and post-15 minutes plasma insulin levels during glucose-stimulated insulin secretion

test in control *Hcfc1*$^{lox/Y}$ (n=10) and knockout *Alb-Cre-ERT2*^{tg} ; *Hcfc1*$^{hepKO/Y}$ (n=10) males 4d post-tamoxifen treatment. (F) Glycemia during insulin tolerance test in control *Hcfc1*$^{lox/Y}$ (n=9) and knockout *Alb-Cre-ERT2*^{tg} ; *Hcfc1*$^{hepKO/Y}$ (n=10) males 4d post-tamoxifen treatment.

Supplementary Figure 4. *Hcfc1*$^{hepKO/Y}$ knockout males present an altered hepatic metabolism by 7d post-tamoxifen treatment. (A) Column graphs showing levels of intermediates of energy metabolism in 0d control liver (suffix WT; n=4) and 7d post-tamoxifen treated knockout *Alb-Cre-ERT2*^{tg} ; *Hcfc1*$^{hepKO/Y}$ male (suffix KO; n=2) livers. The intermediates assayed were aspartic acid (Asp); glutamate (Glu); dihydroxyacetone phosphate (DHAP) and 3-phosphoglycerate (3-PGA); hexose (e.g. glucose; H1); glucose-1-phosphate, glucose-6-phosphate, and fructose-6-phosphate (abbreviated as Hex-P here); fumaric acid (Fum); and succinic acid (Suc). The p-values are indicated in the figure. (B) Column graphs showing levels of acylcarnitines in 0d control liver (suffix WT; n=4) and 7d post-tamoxifen treated knockout *Alb-Cre-ERT2*^{tg} ; *Hcfc1*$^{hepKO/Y}$ male (suffix KO; n=2) livers. The acyclcarnitines assayed were free carnitine (C0); acetylcarnitine (C2); propionylcarnitine (C3); butyrylcarnitine (C4); isovalerylcarnitine/ 2-methylbutyrylcarnitine (C5); glutarylcarnitine/ hydroxyhexanoylcarnitine (C5-DC); methylglutarylcarnitine (C5-M-DC); hydroxyisovalerylcarnitine/ hydroxy-2-methylbutyryl/ methylmalonylcarnitine (C5-OH); caproylcarnitine/ fumarylcarnitine (C6); tetradecanoylcarnitine/ myristylcarnitine (C14); hexadecanoylcarnitine/ palmitoylcarnitine (C16); octadecanoylcarnitine/ stearylcarnitine (C18); and octadecenoylcarnitine/ oleylcarnitine (C18:1). The p-values are indicated in the figure. (C) Column graphs showing levels of bile acids in 0d control liver (suffix WT; n=4) and 7d post-tamoxifen treated knockout *Alb-Cre-ERT2*^{tg} ; *Hcfc1*$^{hepKO/Y}$ male (suffix KO; n=6) livers. The bile acids assayed were cholic acid (CA); glycocholic acid (GCA); α-muricholic acid (MCA(a)); β-muricholic acid (MCA(b)); ω- muricholic acid (MCA(o)), taurocholic acid

(TCA), taurodeoxycholic acid (TDCA) and tauroursodeoxycholic Acid (TUDCA). The p-values are indicated in the figure. (D) Column graphs showing levels of amino acids in 0d control liver (suffix WT; n=4) and 7d post-tamoxifen treated knockout *Alb-Cre-ERT2*^{tg} ; *Hcfc1*$^{hepKO/Y}$ male (suffix KO; n=2) livers. The amino acids assayed were aspartate (Asp); glutamine (Gln); histidine (His); and serine (Ser). The p-values are indicated in the figure.

Supplementary Figure 5. *Hcfc1*$^{hepKO/Y}$ knockout male livers display significant tissue disorganization. Liver histology was assessed by haematoxylin (blue) and eosin (shades of pink) staining of paraffin-embedded sections from control liver (0d), and 4d, 7d, 9d, 11d, 14d, and 18d post-tamoxifen treated knockout *Alb-Cre-ERT2*^{tg} ; *Hcfc1*$^{hepKO/Y}$ male livers. Yellow arrows point to some hepatocytes showing variable nuclear sizes. Dotted white boundaries point to distinct tightly packed hepatocyte clusters. Scale bar: 100 µm.

Supplementary Figure 6. Intracellular glycogen levels are progressively depleted in *Hcfc1*$^{hepKO/Y}$ knockout male livers. Hepatic glycogen content was detected by PAS staining (purple) in paraffin-embedded sections from control liver (0d), and 4d, 7d, 14d, and 18d (two images) post-tamoxifen treated knockout *Alb-Cre-ERT2*^{tg} ; *Hcfc1*$^{hepKO/Y}$ male livers. The sections were also stained with nuclear Mayer's hematoxylin (blue). Scale bar: 100 µm for all.

Supplementary Figure 7. *Hcfc1*$^{hepKO/Y}$ knockout male livers display presence of significant inflammatory infiltrate from 7d post-tamoxifen treatment onwards. Hepatic inflammation was visualized by DAB immunostaining for macrophage marker, F4/80 (brown) in paraffin-embedded sections from control liver (0d), and 7d, 9d, 11d, 14d, and 18d post-tamoxifen treated knockout *Alb-Cre-ERT2*^{tg} ; *Hcfc1*$^{hepKO/Y}$ male livers. Scale bar: 100 µm for all.

Supplementary Figure 8. *Hcfc1*$^{hepKO/Y}$ knockout male livers display progressive build-up of collagen fibers from 9d post-tamoxifen treatment onwards. Hepatic fibrosis was identified by Sirius Red staining (red) of paraffin-embedded sections from control liver (0d), and 7d, 9d, 11d, 14d, and 18d post-tamoxifen treated knockout *Alb-Cre-ERT2*^{tg} ; *Hcfc1*$^{hepKO/Y}$ male livers. Black arrows point to some collagen fibers. Scale bar: 100 μm for all.

Supplementary Figure 9. *Hcfc1*$^{hepKO/Y}$ knockout livers display hepatic steatosis, altered mitochondria morphology and fibrosis. Electron microscopy was performed to examine the ultrastructural details of control liver (0d), and 4d, 7d, and 14d post-tamoxifen treated knockout *Alb-Cre-ERT2*^{tg} ; *Hcfc1*$^{hepKO/Y}$ male liver samples. The regions highlighted by dotted squares in A1, B1, C1, and D1 are shown at higher magnification in A2, B2, C2, and D2, respectively. Mitochondria pointed by black arrows in A2, B2, C2, and D2, respectively can be seen at even higher magnification in A3, B3, C3, and D3, respectively. Black arrowheads in D1 point to perisinusoidal collagen fibers. Yellow arrows in D1-D2 point to vacuoles. Red arrow in D1 points to a lysosome. d, days post-tamoxifen treatment; Gly, glycogen; KO, knockout; L, lipid droplets, Mito, mitochondria; N, nuclei, S, sinusoid. Scale bar: 100 μm.

Supplementary Figure 10. *Hcfc1*$^{hepKO/Y}$ knockout male livers display significantly alterations in levels of mitochondrial-gene-specific RNAs. Heat map of down-regulated (A) and up-regulated (B) mitochondrial-gene-specific RNA levels from 0d to 9d post-tamoxifen treatment in knockout *Alb-Cre-ERT2*^{tg} ; *Hcfc1*$^{hepKO/Y}$ males. The color key, shown beside the heat map, indicates the associated Z-score, with white signifying the lowest score.

Supplementary Figure 11. *Hcfc1*$^{hepKO/Y}$ knockout male livers display up-regulation of genes related to chronic injury. (A) Summary of the results of the functional enrichment analysis on

the transcripts that are up-regulated in *Hcfc1*$^{hepKO/Y}$ knockout males. Only the GO terms displaying an enrichment p-value lower than 10^{-10} were kept for analysis with the REVIGO tool. REVIGO aggregates synonymous GO terms and displays the aggregated terms as circles where the distance among circles indicates their similarity within the GO structure and their color indicates the associated p-value, with blue signifying the lowest p-values. Selected GO terms with highest p-values are shown with the circle aggregates. The scale for p-values is shown beside the REVIGO-representation. (B) The genes up-regulated in knockout male livers were ranked by the fold-change (knockout value minus wildtype value for each time point), and a gene-enrichment analysis was performed using this ranked gene list against the HALLMARKS set (http://software.broadinstitute.org/gsea/msigdb/collection_details.jsp#H). The most statistically significant terms in the up-regulated category were related to inflammatory response, epithelial-to-mesenchymal transition, TNFα signaling via NFKB, IL6/JAK/STAT3 signaling, apoptosis, mitotic spindle, angiogenesis, cholesterol homeostasis, complement, KRAS signalling, estrogen response (early and late), p53 pathway, MTORC1 signaling, hypoxia, IL2/STAT5 signaling, allograft rejection, UV response, myogenesis and apical junction.

Supplementary Figure 12. Plot showing fold-change in expression of HCF-1 bound genes that still retain a significant HCF-1 peak upon loss of HCF-1 at 4d post-tamoxifen treatment in knockout *Alb-Cre-ERT2*^{tg} ; *Hcfc1*$^{hepKO/Y}$ male livers.

Supplementary Figure 13. Mitochondria-specific protein levels are significantly reduced in *Hcfc1*$^{hepKO/Y}$ knockout males by 5d post-tamoxifen treatment. (A) Immunofluorescence analysis of paraffin-embedded sections from control (0d) liver and 7d post-tamoxifen treated knockout *Alb-Cre-ERT2*^{tg} ; *Hcfc1*$^{hepKO/Y}$ male livers stained with DAPI (blue) and antibody

against ATP5α (red) or UQCRC2 (red) or MTCO1 (red). (B) Immunofluorescence analysis of paraffin-embedded sections from control (0d) liver, and 7d and 18d post-tamoxifen treated knockout *Alb-Cre-ERT2*^{tg} ; *Hcfc1*$^{hepKO/Y}$ male livers stained with antibody against OXPHOS. (C) Succinate dehydrogenase (SDH) activity assay on paraffin-embedded sections from control (0d) liver, and 7d and 18d post-tamoxifen treated knockout *Alb-Cre-ERT2*^{tg} ; *Hcfc1*$^{hepKO/Y}$ male livers. Dotted circles point to hepatocyte clusters with higher SDH activity. Scale bar: 50 μm.

Supplementary Figure 14. *Hcfc1*$^{hepKO/+}$ heterozygous females display characteristics typical of mild liver injury by 18d post-tamoxifen treatment. (A) Haematoxylin (blue) and eosin (shades of pink) staining of paraffin-embedded sections from 0d control and 18d post-tamoxifen treated heterozygous *Alb-Cre-ERT2*^{tg} ; *Hcfc1*$^{hepKO/+}$ female livers. (B and C) Immunofluorescence analysis of paraffin-embedded sections from 0d control and 18d post-tamoxifen treated heterozygous *Alb-Cre-ERT2*^{tg} ; *Hcfc1*$^{hepKO/+}$ female livers stained with DAPI (blue) together with antibodies against β-catenin (red) and HCF-1 (green). (D) Hepatic glycogen was visualized by PAS staining (purple) of paraffin-embedded sections from 0d control and 18d post-tamoxifen treated heterozygous *Alb-Cre-ERT2*^{tg} ; *Hcfc1*$^{hepKO/+}$ female livers. The sections were also stained with nuclear Mayer's hematoxylin (blue). (E) DAB immunostaining for macrophage marker, F4/80 (brown) on paraffin-embedded sections from 0d control and 18d post-tamoxifen treated heterozygous *Alb-Cre-ERT2*^{tg} ; *Hcfc1*$^{hepKO/+}$ female livers. The sections were also stained with nuclear Mayer's hematoxylin (blue). (F) Immunofluorescence analysis of paraffin-embedded sections from 0d control and 18d post-tamoxifen treated heterozygous *Alb-Cre-ERT2*^{tg} ; *Hcfc1*$^{hepKO/+}$ female livers stained with DAPI (blue) together with antibody against smooth muscle α-actin (SMA; green). (G) Sirius red staining of paraffin-embedded sections from 0d control and 18d post-tamoxifen treated heterozygous *Alb-Cre-ERT2*^{tg} ; *Hcfc1*$^{hepKO/+}$ female livers. (H) TUNEL assay was performed

on paraffin-embedded sections from 0d control and 18d post-tamoxifen treated heterozygous *Alb-Cre-ERT2*^{tg} ; *Hcfc1*$^{hepKO/+}$ female livers co-stained with HCF-1 (green). TUNEL-positive apoptotic cells are shown in red. Scale bar: 100 μm.

Supplementary Figure 15. *Hcfc1*$^{hepKO/+}$ heterozygous female livers display mild steatosis and transient loss of glycogen. (A) The presence of steatosis was estimated by Oil Red O staining of cryo-sections from control liver (0d), and 7d, 11d, and 18d post-tamoxifen treated heterozygous *Alb-Cre-ERT2*^{tg} ; *Hcfc1*$^{hepKO/+}$ female livers. (B) Hepatic glycogen content was detected by PAS staining (purple) in paraffin-embedded sections from control liver (0d), and 7d, 11d, and 18d post-tamoxifen treated heterozygous *Alb-Cre-ERT2*^{tg} ; *Hcfc1*$^{hepKO/+}$ female livers. The sections were also stained with nuclear Mayer's hematoxylin (blue). Scale bar: 100 μm.

Supplementary Figure 16. *Hcfc1*$^{hepKO/+}$ heterozygous female livers display presence of significant inflammatory infiltrate from 9d post-tamoxifen treatment onwards. Hepatic inflammation was visualized by DAB immunostaining for macrophage marker, F4/80 (brown) in paraffin-embedded sections from control liver (0d), and 7d, 9d, 11d, 14d, and 18d post-tamoxifen treated heterozygous *Alb-Cre-ERT2*^{tg} ; *Hcfc1*$^{hepKO/+}$ female livers. Scale bar: 100 μm for all.

Supplementary Figure 17. *Hcfc1*$^{hepKO/+}$ heterozygous female livers display mild presence of collagen fibers from 9d post-tamoxifen treatment onwards. Hepatic fibrosis was identified by Sirius Red staining (red) of paraffin-embedded sections from control liver (0d), and 7d, 9d, 11d, 14d, and 18d post-tamoxifen treated heterozygous *Alb-Cre-ERT2*^{tg} ; *Hcfc1*$^{hepKO/+}$ female livers. Black arrows point to some collagen fibers. Scale bar: 100 μm for all.

Supplementary Figure 18. HCF-1-positive hepatocytes proliferate and replace HCF-1-negative hepatocytes in *Hcfc1*$^{hepKO/+}$ heterozygous females. (A-B) Immunofluorescence analysis of paraffin-embedded sections from 7d (A) and 18d (B) post-tamoxifen treated heterozygous *Alb-Cre-ERT2*^{tg} ; *Hcfc1*$^{hepKO/+}$ female livers stained with DAPI (blue) and antibodies against HCF-1 (green) and Ki67 (red). White arrows point to HCF-1^{+}- and Ki67^{+}-hepatocytes. d, days post-tamoxifen treatment.

Supplementary Tables

Supplementary Table 1. Raw tag counts on selected *Hcfc1* exons along with the expected-splicing events. The size of the *Hcfc1* exon 1 is 193; exon 2 is 149; exon 3 is 161; and exon 4 is 209 bp.

Supplementary Table 2. List of identified 654 down-regulated genes in *Alb-Cre-ERT2*^{tg} ; *Hcfc1*$^{\mathrm{hepKO/Y}}$ knockout male livers.

Supplementary Table 3. List of identified 521 initially up-regulated genes in *Alb-Cre-ERT2*^{tg} ; *Hcfc1*$^{\mathrm{hepKO/Y}}$ knockout male livers.

Supplementary Table 4. List of identified 2871 later up-regulated genes in *Alb-Cre-ERT2*^{tg} ; *Hcfc1*$^{\mathrm{hepKO/Y}}$ knockout male livers.

Supplementary Table 5. Enriched GO terms in identified 654 down-regulated genes in *Alb-Cre-ERT2*^{tg} ; *Hcfc1*$^{\mathrm{hepKO/Y}}$ knockout male livers.

Supplementary Table 6. Enriched GO terms in identified 521 initially up-regulated genes in *Alb-Cre-ERT2*^{tg} ; *Hcfc1*$^{\mathrm{hepKO/Y}}$ knockout male livers.

Supplementary Table 7. List of total HCF-1 peaks near transcription start site (TSS) or not associated with a TSS (i.e., more than +/- 250 bp) identified by ChIP-seq analysis.

Supplementary Table 8. List of transcription units associated with HCF-1 Peaks within +/- 250 bp of only one gene/transcription unit (unidirectional) and with two or sometimes (but rarely so) more than two transcription units.

Supplementary Table 9. Enriched GO terms in identified 280 HCF-1 bound down-regulated genes in *Alb-Cre-ERT2*^{tg} ; *Hcfc1*$^{hepKO/Y}$ knockout male livers at 4d post-tamoxifen treatment.

Supplementary Table 10. Enriched GO terms in identified 163 HCF-1 bound up-regulated genes in *Alb-Cre-ERT2*^{tg} ; *Hcfc1*$^{hepKO/Y}$ knockout male livers at 4d post-tamoxifen treatment.
